# Vector competence for dengue-2 virus of permethrin resistant/ susceptible *Aedes aegypti* sub-populations from selected sites in Lamu County, Kenya

**DOI:** 10.1371/journal.pone.0359810

**Published:** 2026-10-07

**Authors:** Kulthoom Suhailah Qureish, Francis Mulwa, Richard Odinga, Santos Yalwala, Joel Lutomiah, Joel L. Bargul, Gerald G. Kellar, John Eads, Fredrick Eyase

**Affiliations:** 1 Walter Reed Army Institute of Research-Africa/Kenya Medical Research Institute, Kisumu, Kenya; 2 Centre for Virus Research, Kenya Medical Research Institute, Nairobi, Kenya; 3 Department of Biochemistry, Jomo Kenyatta University of Agriculture and Technology (JKUAT), Nairobi, Kenya; 4 Walter Reed Army Institute of Research-Africa, Nairobi, Kenya; ICMR-National Institute of Malaria Research, INDIA

## Abstract

Dengue virus, primarily transmitted by *Aedes aegypti* mosquitoes, is endemic in Lamu County. There is currently no definitive treatment for dengue; therefore, vector control remains the most effective method to curb transmission. Prolonged exposure of *Ae. aegypti* to insecticides may confer resistance. This study aimed to determine the levels of resistance to permethrin, a commonly used insecticide, and to compare the vectorial competence and genetic differences of resistant versus susceptible populations for dengue-2 virus. *Ae. aegypti* mosquitoes were collected from Lamu (Shela, Hindi, and Mokowe) using traps and assayed for permethrin resistance/ susceptibility. WHO insecticide resistance assays were conducted. The mosquitoes (F_2_) were exposed to dengue-2 infectious bloodmeals and fully engorged mosquitoes selected, incubated for up to 21 days, and tested for infection and dissemination by plaque assay. Individual legs were dissected from 31 female (F_0_) mosquitoes for isolation of genomic DNA. Polymerase Chain Reaction was used to amplify the target mitochondrial DNA using HCO2198 and LCO1490 primers. The amplicons were Sanger sequenced to determine genetic diversity. The Shela sub-population displayed susceptibility (>98% mortality), whereas the Hindi and Mokowe sub-populations exhibited resistance, with mortality rates of 47% and 37.6%, respectively. Of the mosquitoes from Hindi (n = 60); Mokowe (n = 90); and Shela (n = 130), n = 14 (23%), n = 24 (27%), and n = 40 (31%) respectively, had midgut infections with dengue-2; while n = 12 (20%), n = 11 (12%), and n = 21 (16%) respectively had disseminated infection. Genetic analysis showed that all the samples were *Ae. aegypti formosus* sub-species. Haplotype diversity was high at 1.0 while nucleotide diversity was low at 0.01184. These findings suggest *Ae. aegypti formosus* sub-species is prevalent in Lamu County. The study also demonstrated high infection and dissemination rates among the *Ae. aegypti* sub- populations.

## Introduction

*Aedes aegypti* mosquito is the primary vector for dengue virus [1]. It is mainly found in tropical and sub-tropical regions such as sub-Saharan Africa, the South American continent and South Asian countries [2]. It has also spread and managed to establish populations in temperate regions such as parts of Europe and East Asia [3]. Dengue fever is caused by four dengue virus (DENV) serotypes: DENV-1, DENV-2, DENV-3 and DENV-4 [4]. Dengue virus serotype 2 (DENV-2) is the most prevalent in Kenya, particularly along the coastal region [5].

Globally, an estimated 390 million dengue virus infections occur annually, of which 500,000 infected are hospitalized and more than 20,000 result in death [6]. In 2023, 6.5 million cases and over 6,800 deaths attributed to DENV were recorded globally, majority of cases being from South America, and South and Southeast Asian regions [7]. In 2024, the number of reported cases increased to 14.1 million globally, with the number of reported dengue-related deaths being more than 9,508 [8]. More than 3.6 million dengue cases and close to 2000 deaths were reported in 2025 [9].

In Kenya, the first dengue outbreak, which was caused by DENV-2, was reported in 1982 in the coastal towns of Malindi and Kilifi [10]. Since then, there have been subsequent dengue outbreaks in Kenya. There was a DENV-3 outbreak in 2011 in Mandera, which is in northern Kenya, with 381 suspected cases [11]; and a multiple serotypes (DENV-1, DENV-2, and DENV-3) outbreak in Mombasa city in the coastal region between 2013 and 2014 with 267 suspected cases; 101 confirmed and 1 death [12]. In 2017, there was another DENV-2 outbreak along the Kenyan coast which resulted in 1,117 reported cases and one death [13]. In 2019, the Kenyan south coast experienced a DENV-3 outbreak [14] with 286 confirmed cases. A reemergence of dengue occurred in Lamu and Mombasa counties in 2021 with 867 suspected cases; 36 of which were confirmed and 2 deaths [15]. In 2024, there was a multi-country outbreak of dengue which affected several countries in Africa including 13 countries in West Africa and two countries in Eastern Africa: Ethiopia and Kenya. There was a total of 88 cases reported from Kenya by April, 2024 [16].

These high infection rates cause a strain on health systems by overwhelming hospital capacity and healthcare providers. Dengue fever has a negative impact on the economy as well through high medical costs and lost productivity of the infected people.

Currently, there is neither a specific drug treatment for dengue nor a suitable approved vaccine for the general population [17] leaving *Aedes* vector control as the only way to combat this disease. Vector control is achieved by various means, for instance by using insecticide treated nets and screens, and by spraying with insecticides [18]. While nets and screens have proven effective, their impact is increasingly constrained by the rise of insecticide resistance among target vectors, which is caused by a combination of genetic, biochemical, and environmental factors [19]. This resistance reduces their insecticidal efficacy, compounded by sustainability challenges such as wear and tear of the deployed screens over time [20]. The screens/ nets are highly effective against *Anopheles* mosquitoes but less so against other species such as *Culex* and *Aedes* mosquitoes [21]. *Aedes* mosquitoes are dawn and dusk feeders, therefore nets/ screens may not be very effective against them [22]. They may also be too expensive or unavailable to deploy in needy households [23]. This positions insecticides as the primary approach to vector control.

Different types of insecticides are used for vector control including organophosphates such as malathion and P-methyl which inhibit the enzyme acetylcholinesterase (AChE), resulting in overabundance of the neurotransmitter acetylcholine. It accumulates at nerve synapses leading to nervous system overstimulation which causes hyperactivity, paralysis then death [24]. Organochlorines such as dieldrin and dichloro-diphenyl-trichloroethane (DDT) interact with the neuron membrane and alter the transmission of the nerve impulses by opening their sodium ion channels which causes them to fire rapidly leading to convulsive seizures and death [25]. Carbamates like bendiocarb interfere with acetylcholinesterase therefore disrupting the nervous system leading to overstimulation of receptors, paralysis, and death [26]. These groups of insecticides are gradually being replaced by pyrethrins due to their significant and persistent toxic effects on humans and the environment [27]. One such pyrethrin is permethrin, which the present study focuses on.

Extensive use of insecticides and agricultural pesticides has led to development of resistance in many insect species [19]. Permethrin resistance has been observed in some *Ae. aegypti* populations to varying degrees [28,29] probably due to the differences in their genetic makeup [30]. In Lamu County, located on the Kenyan coast, there have been outbreaks of arboviral diseases such as dengue and chikungunya [31], as recently as 2025. It is believed that this is due to ineffective vector control due to insecticide resistance among vector populations [32].

Knockdown resistance (kdr), also known as altered target – site resistance [33] due to the mutation that alters the target- site, reduces or removes the toxic effect of the insecticide [34]. These kdr mutations are passed down through generations. The mode of inheritance of insecticide resistance shows homozygosity of the resistance and susceptible genes involved [35].

Some studies have shown that insecticide resistance may affect the vector competence of *Ae. aegypti* mosquitoes by increasing dissemination rates and altering their gut microbiota [36]. Vector competence is the intrinsic ability of a vector to be infected by a pathogen, amplify, disseminate, and transmit it [37]. This is affected by extrinsic and intrinsic factors [38]. Extrinsic factors include temperature and gut microbiota; higher temperatures increase susceptibility of mosquitoes to viruses [39]. The development of many pathogens is temperature sensitive with higher temperatures decreasing the extrinsic incubation period for the viruses [40]. Microbiota found in the gut of insect vectors also decrease viral and parasitic infections in mosquito vectors by activating immune responses or directly inhibiting pathogen development [41].

Internal factors include the immune responses and barriers such as: midgut infection barrier (MIB) which prevents the virus from attaching to the receptors present on midgut epithelial cells; midgut escape barrier (MEB) which prevents the release of viral particles from midgut epithelial cells; salivary gland infection barrier (SIB) which prevents the virus from infecting acinar cells; and salivary gland escape barrier (SEB) which prevents releasing of viral particles from salivary gland acinar cells [42].

To determine the genetic differences between sub-populations, the Cytochrome c oxidase subunit 1 (CO1) gene has proven very useful [43]. The CO1 gene is considered ideal for barcoding due to its presence in most living eukaryotes [44].

There have been recurring outbreaks of dengue, mostly associated with DENV-2, in most of coastal Kenya since 2011, with limited data on insecticide resistance of *Ae. aegypti* in Kenya, and how this impacts arbovirus transmission. Therefore, this study aimed to investigate the insecticide resistance among *Ae. aegypti* sub-populations in Lamu County and its effect on vector ability to acquire, amplify and transmit DENV-2, as well as entomological factors that contribute to dengue transmission. The findings of this study will help to understand the epidemiology of dengue in the county.

## Materials and methods

### Ethics approval

This study was approved by the Scientific Ethics Review Unit (SERU) of the Kenya Medical Research Institute (KEMRI) under approval number, SERU 4768. Approval of the protocol and use of mice in the study was granted via the Animal Care and Use Committee (ACUC) (KEMRI/ACUC/02.09.23).

The mice were anesthetized during feeding of *Ae. aegypti* mosquitoes using Pentobarbital sodium injection at a dosage of 75 mg/kg, and all efforts were made to minimize suffering. They were sacrificed by injecting them intraperitoneally with Ketamine Hydrochloride at a dosage of 150 mg/kg. The mice were monitored daily over a period of six weeks through the mosquito feedings until they were euthanized.

Informed consent was not required owing to this study not involving human participants.

### Study sites

This study was conducted in Lamu County, along the Kenyan Coast (Fig 1) where three sites were selected for sample collection: Shela, Hindi, and Mokowe. Lamu County (latitude 2.2696° S, longitude 40.9006° E) generally experiences a warm, humid tropical climate with temperatures ranging from 24°C to 30°C throughout the year making it conducive for mosquito breeding. The county has a bimodal pattern of rainfall with the long rains occurring between April and July, with the highest rainfall occurring in the month of May; and short rains in November and December.

**Fig 1:**
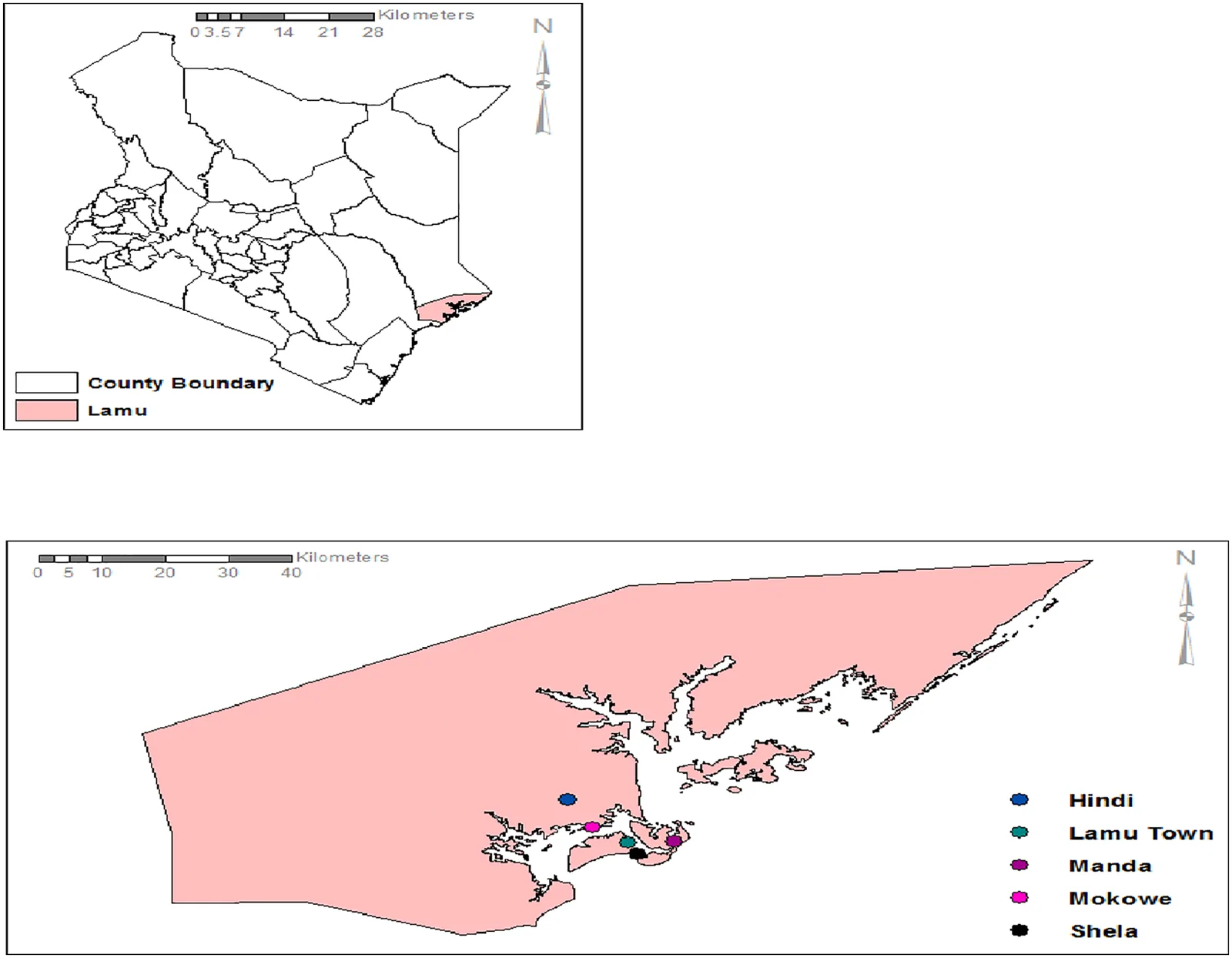
The map of Kenya and, inset, Lamu County showing the sampling sites. Base maps, boundaries and shape files of Kenyan map and administrative boundaries of the County and Sub-county were derived from GADM data version 4.1 (https://gadm.org) and the maps were generated using ArcGIS Version 10.2.2 (http://desktop.arcgis.com/en/arcmap) advanced license courtesy of Samuel Owaka.

### Collection and identification of mosquito samples

Adult mosquitoes were sampled using BG-Sentinel 2 Traps and CDC light traps baited with carbon dioxide (CO_2_) [45,46]. The mosquitoes were collected during periods of higher population densities which usually coincide with rainy seasons, that is, April – June of 2023 (S1 Table, S1 and S2 Files). The traps were set outdoors close to potential breeding grounds like stagnant water and vegetation, between 6.30 pm and 7:00 pm in the evening and collected the following morning between 6:00am and 6.30am. The trapped mosquitoes were knocked down using 50% trimethylamine [47] and transported in mesh cages to the KEMRI laboratories in Kisumu for morphological identification to species level using taxonomic keys [48,49].

### Genetic diversity determination

#### DNA extraction.

Field collected (n = 31) female mosquitoes (F_0_) were sampled from various collection sites in Shela (n = 11), Hindi (n = 10), and Mokowe (n = 10). Each mosquito was from a different site to avoid analyzing siblings. Individual mosquito legs [50] were dissected using sterile forceps and placed in a sterile RNase/DNase-free Eppendorf tube containing a sterile copper bead, 200 ml of Phosphate buffered saline was added into each Eppendorf tube, and the sample homogenized in an Omni Bead Rapture 24 (OMNI International) set to run at the speed of 2.10 mm for 20 seconds. Qiagen™ DNeasy Blood and Tissue Extraction Kit [51] was then used to extract DNA from the homogenates following the manufacturers directives.

#### Polymerase chain reaction.

Polymerase Chain Reaction (PCR) was used to amplify the target mitochondrial DNA (COI) in mosquito samples using the universal reverse primer HCO2198 and forward primer LCO1490. The PCR master mix was prepared in a Biosafety level II molecular laboratory and contained 12.5 µl Amplitaq solution, 1 µl forward primer, 1 µl reverse primer, and 7.5 µl sterile nuclease free water. Next, 22µl of the master mix was transferred into well-labelled RNase-free, 0.2 ml (8-strip format, Invitrogen) PCR tubes and 3 µl of sample DNA added into each tube. The tubes were tightly sealed, vortexed, and placed in an Eppendorf Thermocycler set to run 35 reaction cycles of 94°C for 30 secs, 55°C for 30s, and 72°C for 60s. The amplicons were visualized in a 2% agarose gel with an expected band size of approximately 650 bp, stained with SYBR Green. The amplicons underwent clean up to remove leftover primers and deoxyribonucleotide triphosphates (dNTPs) from PCR. This was done using the Exo-CIP™ Rapid PCR Cleanup protocol [52]. The products were then quantified to determine the concentration of DNA using a nanodrop spectrophotometer, with an expected threshold of 50ng [53], and Sanger sequenced [54].

### Bioassays for determination of susceptibility and resistance of *Ae. aegypti* to permethrin

*Aedes aegypti* mosquitoes were subjected to World Health Organization (WHO) tube bioassays for susceptibility to permethrin [55].

Twenty-five mosquitoes were aspirated at a time from a total of one hundred from each of the three sites (n = 300) and transferred into a holding tube to ensure that damaged specimens were not included in the test. The holding tubes were set upright, mesh-screen end uppermost for approximately 30 minutes after which, any mosquitoes that may have been damaged during aspiration were removed. A sheet of paper impregnated with 0.75% permethrin insecticide was introduced into each of the exposure tubes then rolled into a cylinder and fastened into position with a copper clip. This dose is not the recommended one for evaluating the susceptibility of *Aedes* mosquitoes, however, it is a commonly used one [56–58]. The recommended WHO permethrin dose for *Aedes* mosquitoes is 0.4% [59].

The mosquitoes were introduced into the exposure tube by attaching it to a vacant screw-top in the slide. The mosquitoes were gently blown down into the exposure tube. The slide was closed and the holding tube detached. The exposure tubes were left standing upright with mesh-screen end uppermost for 1 hour at 27 °C ± 2 °C and 75% ± 10% relative humidity. The behavior of the mosquitoes was monitored and the number of those knocked down recorded after every 10 minutes for 1 hour.

At the end of the 1 hour exposure period, the mosquitoes were transferred to the holding tubes set on the slide and a pad of cotton wool soaked in 10% sugar solution placed on the mesh-screen. The holding tubes were kept for 24 hours (recovery period) in a secluded, shady place where the temperatures did not exceed 30 °C. At the end of recovery period, (24-hour post-exposure), the number of knocked down or dead mosquitoes was counted and recorded. An adult mosquito was considered to be alive if it was able to fly regardless of the number of legs remaining. Affected specimens that were unable to walk or fly in a coordinated manner were considered knocked down.

#### Calculation and interpretation of mortality rates.

After 24-hour post-exposure, the number of dead mosquitoes in the holding tube was counted. The mortality is calculated by summing the number of dead mosquitoes per site (i.e., out of a total of 100 mosquitoes from four replicates of 25 mosquitoes each per site) expressed as a percentage of the number of exposed mosquitoes using the formula:


$$\begin{array}{c} Mortality=\frac{Total\text{\textasciitilde{}}number\text{\textasciitilde{}}of\text{\textasciitilde{}}dead\text{\textasciitilde{}}mosquitoes}{Total\text{\textasciitilde{}}number\text{\textasciitilde{}}of\text{\textasciitilde{}}Samples}X\text{\textasciitilde{}}100. \end{array}$$

The World Health Organization (WHO, 2022) interprets the insecticide bioassay results as follows: a vector population is considered to be resistant to an insecticide if mortality within the treatment group is < 90%; if the observed mortality within the treatment group is ≥ 90% but <98%, the presence of resistance is possible but not confirmed; a vector population is considered to be susceptible to an insecticide if the mosquito mortality within the treatment group is ≥ 98% [59].

The validity of the bioassay was assessed using control mortality, sample size, and adherence to standard operating procedures (SOPs). WHO (2022) interprets control mortality as follows: if the mortality is < 5%, the test is valid; if it is 5–20%, the test is valid, but mortality in treated groups must be corrected using Abbott’s formula; if it is > 20%, the test is invalid and must be discarded and repeated. For sample size, WHO (2022) recommends testing at least 100 mosquitoes per insecticide (typically four replicates of 20–30 mosquitoes each). Interpretation of resistance status is considered reliable only when this minimum sample size is achieved. The assay was done in accordance with the 2022 WHO insecticide resistance monitoring protocol hence adhered to the SOP [59].

### Vector competence for dengue-2 determination

#### Mosquito rearing.

Mosquitoes were reared in the KEMRI insectary, maintained at a temperature of 28 °C - 32 °C, 70% − 80% relative humidity (RH) and 12:12 hour light: dark (L: D) photoperiod. The batches of F_1_ eggs were dispensed in water on larval trays for hatching in a level 2 insectary. The larvae were fed on Tetramin^®^ fish food until they pupated, then pupae were collected every morning and put in a holding cup containing water. The cups with pupae were placed in a plastic cage with a netting material on top and allowed to develop into adults.

The emerging adults were inactivated by placing them in a −20 °C freezer for one minute, then morphologically identified under a dissecting microscope using taxonomic keys to ensure they are *Ae. aegypti* mosquitoes. The mosquitoes were fed on a diet of 10% glucose solution for 3 days. On the fifth day, they were fed on mice after being starved for 12 hours.

Mice were considered appropriate for this study as fresh blood is preferred by female mosquitoes to lay eggs and they have many similarities to humans in terms of anatomy, physiology and genetics. They respond similarly to humans when undergoing treatment and are also cost effective in terms of housing and feeding [60].

Albino mice were anaesthetized using Pentobarbital sodium injection at a dosage of 75 mg/kg [61]. The mice were injected intraperitoneally then placed in mosquito cages for 30 minutes. Ovicups half-filled with water and lined with ovipapers were placed in the cages for the mosquitoes to lay F_2_ eggs which were then hatched and reared into adulthood.

#### Dengue virus amplification and quantification.

Live preserved viral isolates (DENV-2 008/01/2012) from Mombasa County were amplified in a confluent monolayer of Vero E6 cells in a T-25 flask containing maintenance media and incubated at 37 °C for 7 days. Upon showing signs of cytopathic effect (CPE), the virus was harvested, aliquoted into cryovials and stored at −80 °C for further use.

Quantification of dengue virus was performed by plaque assay, i.e., tenfold serial dilutions of the amplified DENV [62]. Maintenance media (900µl) was added into nine Eppendorf tubes, 100µl of the amplified DENV-2 were transferred to the first Eppendorf tube and pipette mixed with the media, then 100µl of the mixture was picked and transferred to the next Eppendorf tube and pipette mixed. This step was repeated until the virus had been diluted ten times.

The first well of a 12-well plate was inoculated with 100µl of virus over the confluent monolayers of Vero E6 cells, with 100µl of the subsequent dilutions inoculated into the other wells, leaving two wells for the negative controls. The plate was incubated for 1 hour at 37 °C with rocking every 15 minutes. Methylcellulose was added onto the inoculated cells and the plate observed over the next 14 days.

The plate was then fixed for 2 hours with 10% formalin then stained overnight with 0.5% crystal violet. The plaques were counted and calculated to quantify the virus using the formula [63]:


$$\begin{array}{c} \frac{Number\text{\textasciitilde{}}of\text{\textasciitilde{}\textasciitilde{}}plaques}{d\times V}=pfu/ml \end{array}$$

where d is the dilution factor and V is the volume of diluted virus added to the well.

#### Preparation of blood and virus mixture (infectious bloodmeal).

Sheep blood was acquired from a farm in Kabete, collected into 50 ml falcon tubes and transported to the laboratory. Cryovials of the amplified and quantified DENV-2 virus were thawed on ice. Two mice were sacrificed by injecting them intraperitoneally with Ketamine Hydrochloride at a dosage of 150 mg/kg. The mice were skinned carefully with the fatty tissues being removed from the skin. The skins were fitted onto Hemotek columns and secured with bands.

The DENV-2 was mixed with the blood by pipetting in a ratio of 1:1. The mixture was then transferred to the Hemotek feeding column.

#### Mosquito exposure to infectious bloodmeal.

Three experimental replicates were carried out in which mosquitoes (n = 280) were starved of glucose for 12 hours prior to exposure to DENV-2 infectious bloodmeal. The replicates consisted of: 1st replicate (n = 90); 2nd replicate (n = 100); and 3rd replicate (n = 90) *Ae. aegypti* mosquitoes. The samples from Shela were 130 (1st replicate (n = 35), 2nd replicate (n = 50), 3rd replicate (n = 45)); while 90 (1st replicate (n = 30), 2nd replicate (n = 30), 3rd replicate (n = 30)) were from Mokowe; and 60 (1st replicate (n = 25), 2nd replicate (n = 20), 3rd replicate (n = 15)) from Hindi sites, respectively.

They were allowed to feed for 45 minutes. Fully engorged mosquitoes were aspirated into separate cages, counted and incubated for 7, 14 and 21 days post exposure (dpe). They were later fed on 10% glucose solution ad libitum. The three periods of extrinsic incubation (7, 14 and 21 dpe) were chosen to represent early, mid and late phases of virus dissemination kinetics [64].

There were three vector competence indices: (i) the proportion of mosquitoes exposed to infectious bloodmeal that developed midgut infection, (ii) the proportion of mosquitoes with midgut infection that developed a disseminated infection and (iii) the infectious titre of disseminated virus. The last two indices represent two successive aspects of the infection process in mosquitoes that lead to their ability to transmit the virus.

#### Dissection of mosquitoes.

After 7, 14 and 21 dpe, a third of the mosquitoes were sampled from the cage into a container and cold-anaesthetized at −20 °C for 45 seconds and maintained on wet ice (+4 °C). Scalpels were used to pick the mosquitoes by their wings and to hold them in place while separating the legs from the body.

The dissected bodies and legs were placed into separate 1.5 mL microfuge tubes (Eppendorf) containing 500μl and 200µl respectively, of homogenization media (HM), made of MEM, supplemented with 15% FBS, 2% L-glutamine, and 2% antibiotic/antimycotic, then stored in −80 °C freezer to await testing.

#### Test for infection and dissemination rates.

Mosquito bodies were homogenized using a mini bead beater with the aid of a copper bead (BB-caliber airgun shot). They were centrifuged at 8000 rpm for 10 minutes at 4 °C. The supernatants were then inoculated onto a 70% confluent monolayer of Vero cells on 24- well plates. One hundred microliters (100µl) of the abdominal homogenates were added to each of ten wells of the 12-well plate to infect the cells with the remaining two wells used for negative control.

The plates were incubated at 37 °C in a 5% carbon dioxide (CO_2_) incubator with frequent agitation after every 15 minutes for 1 hour to allow for virus adsorption. The infected cell monolayers were then overlaid with 1 ml of maintenance media. The plates were incubated at 37 °C and 5% CO_2_ for 14 days and observed for signs of cytopathic effect (CPE). On observation of CPE, the plates were fixed for 1 hour with 10% formalin, and stained for 2 hours with 0.5% crystal violet, washed on running tap water, dried overnight and the plaques observed on a light box.

The DENV-2 positive bodies were used to determine the infection rates. The legs of the corresponding mosquito bodies that showed signs of CPE underwent the same procedure. Plaques were counted and calculated to determine the viral titer. If the virus was detected in the mosquito’s body but not in the legs, the mosquito was considered to have a non-disseminated infection, limited to the midgut. Detection of virus in the body and legs was considered evidence of successful infection and dissemination, respectively [65].

### Data management and analysis

#### Genetic diversity analysis.

The raw sequences were viewed using Chromas v2.6.6 (Technelysium) [66], edited by trimming and deleting poor-quality sections and converted into FASTA format sequences [67]. Basic Local Alignment Search Tool (BLAST) was used to compare the sequences against the GenBank database to confirm species identity [68]. The sequences were aligned using Molecular Evolutionary Genetics Analysis (MEGA) software via the Muscle algorithm [69].

For genetic diversity analysis, DnaSP v6.12 [70] was used to determine nucleotide variation within the populations and provide information on nucleotide diversity (π), the number of haplotypes (H), and haplotype diversity (Hd). Neutrality tests (Tajima’s and Fu and Li’s tests) were also conducted. The aligned sequences were used to carry out phylogenetic analysis. Sequences belonging to *Ae. aegypti aegypti* (AF390098, AY432106), *Ae. aegypti formosus* (AY056597) and *Ae. albopictus* (MF148303) were downloaded from GenBank and used as references. The combined sequences were aligned in MEGA and used to infer a maximum likelihood phylogeny in IQTREE [71]. The phylogenetic tree was visualized in FigTree v1.4.4 [72].

#### Vector competence.

Data was stored in Microsoft Excel spreadsheet [73] and checked for errors and inconsistencies prior to analysis. Key rates were defined as follows: i) Infection rate (IR) which was determined as the proportion of mosquitoes whose bodies were infected with dengue virus, and ii) Dissemination rate (DIR) as the proportion of mosquitoes whose legs were infected with dengue virus. The proportions (IR and DIR) were calculated and the 95% confidence intervals (CIs) determined using binomial regression model. Proportion test of differences was used to determine significant differences between sites and incubation periods, and the associated p-values determined at 0.05 level of significance. Further, a comparative bar graph was used to compare the rates between the three sites. All the analyses were carried out using STATA version 15.1 [74]. All visualizations were done in RStudio version 2023.06.1 [75].

## Results

### Genetic diversity

There was a high level of genetic diversity between Hindi, Mokowe and Shela towns of Lamu County. Overall, 14 sequences of *Ae. aegypti* mosquitoes showed 14 unique haplotypes with a high haplotype diversity (Hd) of 1.0 (Hd > 0.5). For the individual sites: Hindi sub-population showed 6 haplotypes with Hd of 1.0; Mokowe sub-population showed 6 haplotypes with Hd of 1.0; and Shela sub-population showed 2 haplotypes with Hd of 1.0.

Overall, nucleotide diversity (π) was low (π < 0.5) at 0.01184 with 0.00982 observed for Hindi sub-population and 0.01266 for Mokowe (Table 1). However, this could not be calculated for the Shela sub-population due to the limited number (n = 2) of sequences against the requird minimum of three.

**Table 1:** Genetic diversity of *Ae. aegypti* mosquitoes from Lamu County.

Sites	n	Hn	Hd	π	D	Fu’s F
**Hindi**	6	6	1.0	0.00982	−0.65366	−1.734
**Mokowe**	6	6	1.0	0.01266	−0.76939	−1.298
**Shela**	2	2	1.0	–	–	–
**Combined**	14	14	1.0	0.01184	−1.09398	−5.526

n = Number of sequences, Hn = Number of haplotypes, Hd = Haplotype diversity, **π** = Nucleotide diversity, D = Tajima’s D, Fu’s F = Fu’s selection test.

### Neutrality analysis

Tajima’s D and Fu’s F neutrality tests were carried out to analyse evolutionary history of the *Ae. aegypti* sub-populations. Tajima’s D values were negative overall (−1.09398), in Hindi (−0.65366), and Mokowe (−0.76939). Fu’s F values were also negative: −1.734 in Hindi, −1.298 in Mokowe, and −5.526 for the combined sub-populations (Table 1). These observations align with Tajima’s D test.

### Phylogenetic analysis

Phylogenetic analysis of the mosquito sequences showed that all the *Ae. aegypti* sub-populations were *Ae. aegypti formosus (Aaf)* (Fig 2).

**Fig 2:**
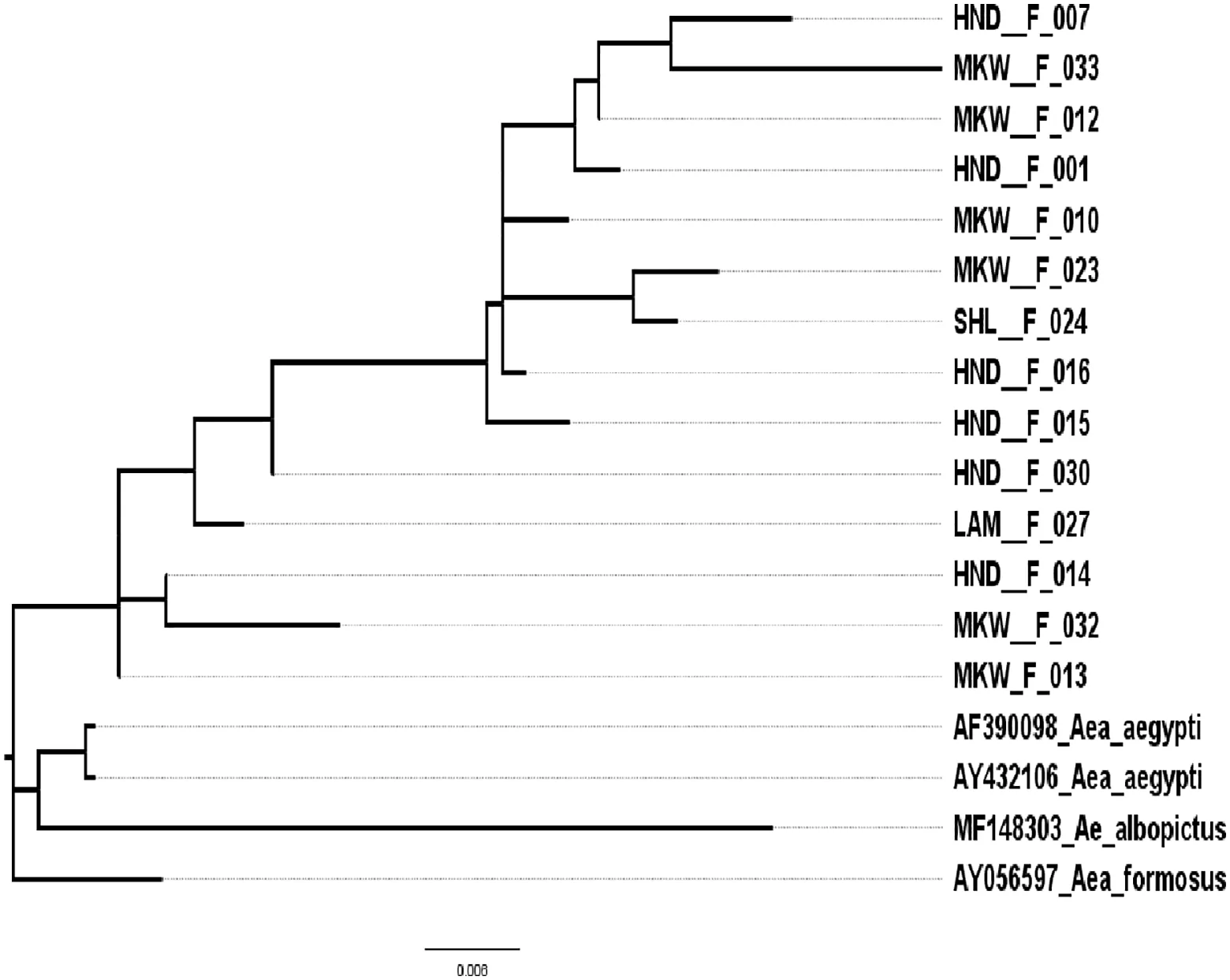
Maximum likelihood tree of *Ae. aegypti* mosquitoes collected from Lamu County.

### Bioassay for determining susceptibility or resistance of *Ae. aegypti* to permethrin

The assayed *Aedes aegypti* mosquitoes were divided into two categories: susceptible (≥ 98% mortality), and resistant (< 90% mortality), in accordance with WHO 2022 guidelines [59].

The Hindi sub-population showed a mortality rate of 46.98% (47%) which is 53% resistance against permethrin; Mokowe sub-population showed a mortality rate of 37.59% (37.6%) which is 62.4% resistance; and the Shela sub-population showed a mortality rate of 98.93% (99%) which is a 1% resistance.

The Shela sub-population displayed significantly lower resistance (1%) compared to the Hindi and Mokowe sub-populations at 53% and 62.4%, respectively (Chi-square = χ² = 458.47, df = 2, p < 0.001; pairwise comparisons, p < 0.05 for all pairs).

The control mortality rate was 3.6%, indicating validity of the bioassay.

### Quantification of dengue virus

The amplified DENV-2 virus had a titre of 6.7 X 10^4^ Pfu/ml while the infectious blood meal’s titre was 6.3 X 10^4^ Pfu/ml. These two titres were fairly comparable.

### Vector competence

#### Infection rate.

The overall infection rate of all three sites was 27.9% (95% CI: 23.0–33.6) and increased with the incubation period from day 7 (5.6%; 95% CI: 2.4–13.0), day 14 (17.0%; 95% CI: 11.0–26.2) and peaking at day 21 (62.2%; 95% CI: 53.0–73.1). The proportion test of difference revealed that the infection rates significantly increased by 56.6% at day 21 (Diff = 0.566, p < 0.001) and by 11.4% at day 14 (Diff = 0.114, p = 0.014), compared to day 7 (Tables 2 and 3).

**Table 2:** Comparison of the infection and dissemination rates in the three study sites.

	Proportion of mosquitoes infected with Dengue virus % (95%CI); n = 280	
	**Body (Infection)**	**Leg (Dissemination)**
**Overall**	27.9 (23.0–33.6); n = 78	15.7 (12.0–20.6); n = 44
**Site**		
Hindi (n = 60)	23.3 (14.7–36.9); n = 14	20.0 (12.1–33.2); n = 12
Mokowe (n = 90)	26.7 (18.9–37.6); n = 24	12.2 (7.0–21.3); n = 11
Shela (n = 130)	30.8 (23.8–39.8); n = 40	16.2 (10.9–23.9); n = 21
Proportion test of the difference between Hindi and Mokowe (diff, p value)	Diff = −0.034, p = 0.639	Diff = 0.078, p = 0.194
Proportion test of the difference between Hindi and Shela (diff, p value)	Diff = −0.075, p = 0.287	Diff = 0.038, p = 0.521
Proportion test of the difference between Mokowe and Shela (diff, p value)	Diff = −0.041, p = 0.511	Diff = −0.04, p = 0.408

**Table 3:** Comparison of infection and dissemination rates in the three study sites by incubation periods.

Site	Day	Body (Infection)	Leg (Dissemination)
**Overall**	Day 7	5.6 (2.4–13.0); n = 5/90	4.4 (1.7–11.6); n = 4/90
	Day 14	17.0 (11.0–26.2); n = 17/100	10.0 (5.6–18.0); n = 10/100
	Day 21	62.2 (53.0–73.1); n = 56/90	33.3 (24.9–44.6); n = 30/90
	Proportion test of difference at day 14 (diff, p value)	Diff = −0.114, p = 0.014*	Diff = −0.056, p = 0.140
	Proportion test of difference at day 21 (diff, p value)	Diff = −0.566, p < 0.001*	Diff = −0.289, p < 0.001*
**Hindi**	Day 7	8.0 (2.1–30.2); n = 2/25	8.0 (2.1–30.2); n = 2/25
	Day 14	10.0 (2.7–37.2); n = 2/20	5.0 (0.7–33.8); n = 1/20
	Day 21	66.7 (46.6–95.3); n = 10/15	60.0 (39.7–90.7); n = 9/15
	Proportion test of difference at day 14 (diff, p value)	Diff = −0.02, p = 0.815	Diff = 0.03, p = 0.689
	Proportion test of difference at day 21 (diff, p value)	Diff = −0.59, p < 0.001*	Diff = −0.52, p < 0.001*
**Mokowe**	Day 7	6.7 (1.7–25.4); n = 2/30	3.3 (0.4–22.9); n = 1/30
	Day 14	26.7 (14.7–48.3); n = 8/30	13.3 (5.4–33.2); n = 4/30
	Day 21	46.7 (31.8-68.4); 14/30	20.0 (9.8–40.9); n = 6/30
	Proportion test of difference at day 14 (diff, p value)	Diff = −0.2, p = 0.039*	Diff = −0.1, p = 0.153
	Proportion test of difference at day 21 (diff, p value)	Diff = −0.4, p < 0.001*	Diff = −0.17, p = 0.039*
**Shela**	Day 7	2.9 (4.1–19.7); n = 1/35	2.9 (0.4–19.7); n = 1/35
	Day 14	14.0 (7.0–27.8); n = 7/50	10.0 (4.4–23.0); n = 5/50
	Day 21	71.1 (59.0–89.7); n = 32/45	33.3 (22.1–50.4); n = 15/45
	Proportion test of difference at day 14 (diff, p value)	Diff = −0.11, p = 0.088	Diff = −0.07, p = 0.217
	Proportion test of difference at day 21 (diff, p value)	Diff = −0.68, p < 0.001*	Diff = −0.3, p < 0.001*

Infection rates were highest in Shela 30.8% (95% CI: 23.8–39.8), followed by Mokowe 26.7% (95% CI: 18.9–37.6) and least in Hindi 23.3% (95% CI: 14.7–36.9).

Of the mosquitoes that were exposed to DENV-2, Hindi (n = 60); Mokowe (n = 90); and Shela (n = 130), n = 14 (23%), n = 24 (27%) and n = 40 (31%) from Hindi, Mokowe and Shela respectively, had midgut infections (Fig 3). However, the observed differences were not significant: (Chi-square χ² = 1.22, df = 2, p = 0.542).

**Fig 3:**
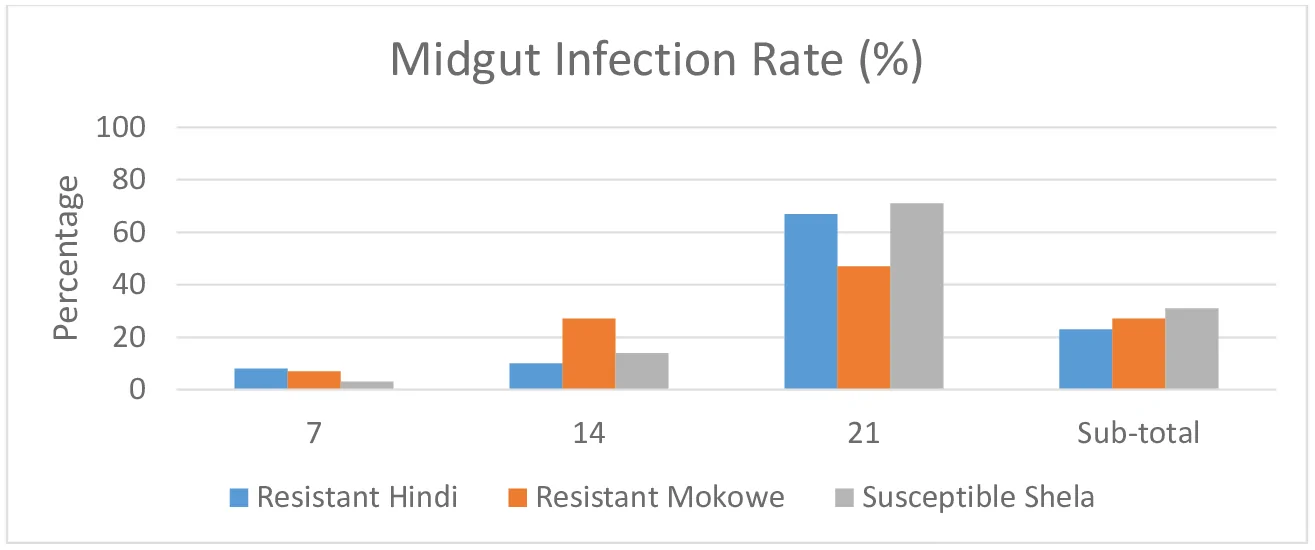
Infection percentages by site and incubation periods.

#### Dissemination rate.

Overall, results showed mosquito dissemination rate of 15.7% (95% CI: 12.0–20.6). The overall dissemination rate increased with the incubation period, the lowest rate observed on day 7 (4.4%, 95% CI: 1.7–11.6), increasing to 10.0% (95% CI: 5.6–18.0) on day 14 and peaking on day 21 (33.3%, 95% CI: 24.9–44.6) post exposure. A proportion test of difference revealed that the dissemination rates significantly increased by 28.9% at day 21 (Diff = 0.289, p < 0.001), while no significant increase was observed at day 14.

By sites, the mosquito dissemination rate was highest in Hindi at 20.0% (95% CI: 12.1–33.2), followed by Shela at 16.2% (95% CI: 10.9–23.9) and the least in Mokowe at 12.2% (95% CI: 7.0–21.3). A proportion test of difference for each site indicated that the dissemination rates significantly increased by 52.0% (Diff = 0.52, p < 0.001), 30.0% (Diff = 0.30, p < 0.001), and 17.0% (Diff = 0.17, p = 0.039) in Hindi, Shela, and Mokowe sites respectively at day 21 compared to day 7. There was no significant increase in dissemination rates in any of the sites at day 14 compared to day 7 (Tables 2–3, and Fig 4).

**Fig 4:**
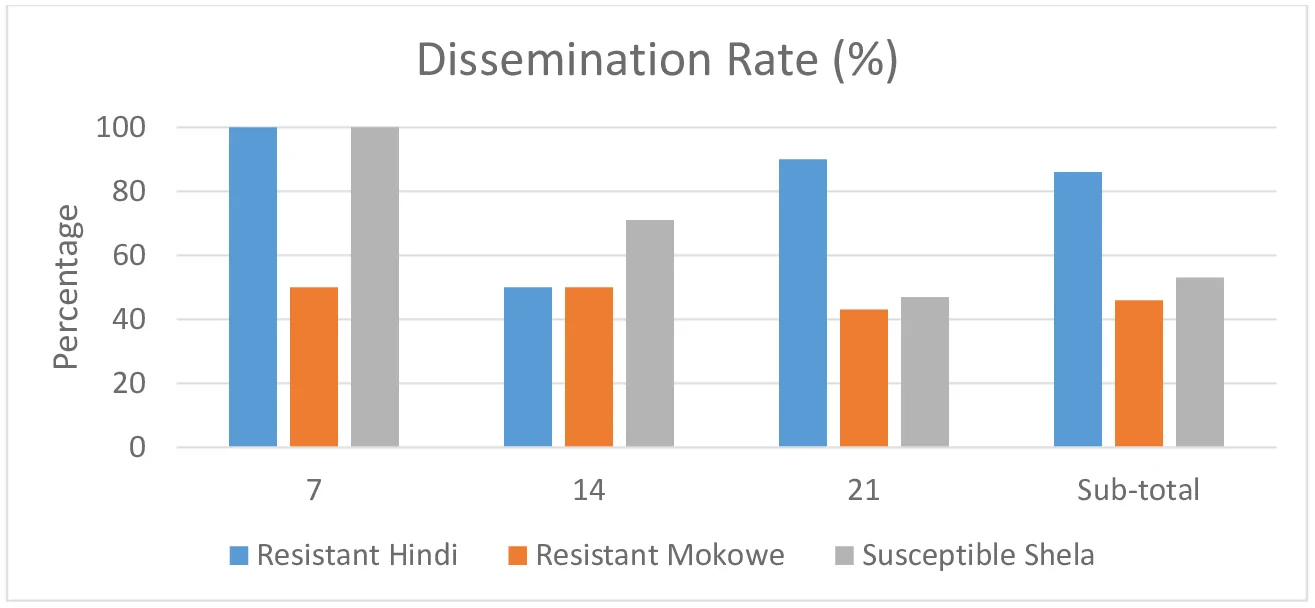
Dissemination percentages by site and incubation periods.

Of the mosquitoes, from Hindi (n = 60); Mokowe (n = 90); and Shela (n = 130) that were exposed to DENV-2, n = 12 (20%), n = 11 (12%), and n = 21 (16%) respectively had disseminated infections. The observed differences were not significant: (Chi-square χ² = 1.68, df = 2, p = 0.432).

Dissemination was used as proxy for transmission rates as saliva collection via capillary method tends to underestimate virus transmission. The results for the transmission rate were therefore the same as those of the dissemination rate.

The plaque assay performed on the positive mosquito bodies and legs confirmed the presence of live viral particles. The mean body and leg titres for all three sites on 7, 14 and 21 days post exposure are visualized on Tables 4 and 5.

**Table 4:** Mean DENV-2 titres (Pfu/ml) in mosquito bodies for all three sites 7, 14 and 21 days post exposure.

Site	Day 7	Day 14	Day 21
**Hindi**	3.2 X 10^4^	3.2 X 10^4^	3.8 X 10^4^
**Mokowe**	3.2 X 10^4^	3.3 X 10^4^	3.5 X 10^4^
**Shela**	3.0 X 10^4^	3.1 X 10^4^	3.8 X 10^4^

**Table 5:** Mean DENV-2 titres (Pfu/ml) in mosquito legs for all three sites 7, 14 and 21 days post exposure.

Site	Day 7	Day 14	Day 21
**Hindi**	2.9 X 10^4^	2.9 X 10^4^	3.2 X 10^4^
**Mokowe**	2.7 X 10^4^	2.9 X 10^4^	2.9 X 10^4^
**Shela**	2.5 X 10^4^	2.6 X 10^4^	2.9 X 10^4^

## Discussion

According to Grant and Bowen (1998), values greater than 0.5 are considered high while those less than 0.5 are considered low when classifying the genetic diversity of populations based on mtDNA haplotype and nucleotide diversity [76]. This has therefore resulted in four key categories: low haplotype diversity (Hd) and low nucleotide diversity (π); high Hd and low π; low Hd and high π; and high Hd and high π [50].

The findings of this study fall within the high haplotype diversity (1.0) and low nucleotide diversity (0.01184) classification which indicate a recent population growth from a small one with few, related ancestors [77]. The new individuals inherited the existing haplotypes in increasing numbers, leading to high haplotype diversity [78]. Gene flow from populations that are genetically close to the population they are flowing to can also cause an increase in haplotype diversity [79]. This increase happened in a short time, however, and hindered the mutation rate and divergence of the haplotypes at nucleotide level, leading to low nucleotide diversity [80].

Two neutrality tests were also carried out: Tajima’s D and Fu’s F tests. Tajima’s D test determines whether a DNA sequence has evolved randomly (neutrally) or non-randomly (selectively). It is done by getting the difference between the mean of pairwise differences and the number of segregating sites [81]. The two values are expected to be the same in a population that has evolved neutrally and maintained a constant size. Deviation from this neutrality and the difference in the values informs us of the population’s history. A negative Tajima’s D value indicates an abundance of rare alleles, most likely caused by an expanding population after a recent bottleneck and selective sweep [82].

Fu’s F test works hand in hand with Tajima’s D test. It also tests for deviation from neutrality by investigating non-random recombination. It is determined by calculating the distribution of alleles and haplotypes in a DNA sequence [83]. Negative Fu’s F values also indicate a recent population expansion and positive selection [84].

The evolutionary history was inferred using the Maximum Likelihood method and Tamura-Nei model [85]. The tree with the highest log likelihood (−3082.37) was shown. Initial trees for the heuristic search were obtained automatically by applying Neighbor-Join and BioNJ algorithms to a matrix of pairwise distances estimated using the Tamura-Nei model and then selecting the topology with superior log likelihood value. The evolutionary analyses were determined in MEGA11 [86].

The phylogenetic analysis revealed that all the samples belonged to the *Ae. aegypti formosus (Aaf)* sub-species which is native to Kenya [87]. *Ae. aegypti formosus* is primarily sylvan (forest- dwelling mosquito), though it has spread into urban areas [88] and become domesticated [89]. *Ae.* *aegypti formosus* is also a known zoophilic mosquito, although this may be changing with its spread into areas densely-populated by humans [90]. This adaptability is a cause for concern as it was previously considered less of a threat than its counterpart *Ae. aegypti aegypti*.

A sample size of thirty-one was considered to be adequate as studies have shown that a minimum sample size of 10–30 individuals per population is adequate for assessing genetic diversity in *Ae. aegypti* using CO1 (cytochrome c oxidase subunit 1) mitochondrial DNA to ensure accurate estimation of haplotype and nucleotide diversity [50,91,92].

*Aedes aegypti* mosquitoes from Shela were 99% susceptible to permethrin insecticide while the Hindi and Mokowe populations were 53% and 62.4% resistant, respectively. This suggests that permethrin is an effective insecticide in Shela, but ineffective in controlling *Ae. aegypti* populations in Hindi and Mokowe. Therefore, these areas need a more effective product to replace permethrin in the control of *Ae. aegypti* vectors.

The midgut infection rate (MIR) for all three sub-populations gradually increased over the three-week duration. A higher viral titre (6.3 X 10^4^ Pfu/ml) in the infectious bloodmeal increases the chance of overcoming the MIB and reducing the time taken for the vector to be infected [93]. Tables 4 and 5 above show an overall increase in the mean dengue-2 viral titre of the *Ae. aegypti* bodies and legs over the incubation period. This suggests that life expectancy of an infected mosquito vector is important for transmission of DENV-2 to occur, since the longer it lives the more the MIB is overcome. There was no significant difference in infection rates between the different populations (Chi-square χ² = 1.22, df = 2, p = 0.542) suggesting that susceptibility/ resistance of *Ae. aegypti* to permethrin has no effect on mosquito susceptibility to DENV-2 infection.

The dissemination rates (DIR) were high for all three sub-populations. This indicates that the midgut escape barrier (MEB) was very vulnerable to virus penetration which was therefore easily released from the midgut epithelial cells. Some studies suggest that the MEB may be affected by factors such as the degree of virus replication or viral load [94]. The DIR seems to be unaffected by permethrin resistance or susceptibility as it varied indiscriminately among the three sub-populations and the days post exposure.

Dissemination was used as proxy for transmission. Saliva collection via capillary method requires forced salivation which tends to underestimate virus transmission rates. The transmission rates in a natural setting are more likely to be higher than in a laboratory setting. Dissemination is considered adequate in predicting the vector’s transmission abilities [95].

The p-values for the infection rate (p = 0.542) and the dissemination rate (p = 0.432) for the three sites; as well as those of the proportion tests of difference between the three sites when compared to each other (p = 0.639, p = 0.287, p = 0.511); were above the 0.05 level of significance. This is statistically not significant and leads to the conclusion that there is no relationship between permethrin resistance/ susceptibility and vector competence.

## Conclusion

The analysis showed that *Ae. aegypti* mosquito sub-populations from Lamu County have high infection and dissemination rates with dengue virus. Although the rates increased significantly with the incubation periods, they varied insignificantly by mosquito collection sites.

The genetic diversity tests confirmed the presence of *Ae. aegypti formosus* in Lamu County. The negative Tajima’s D and Fu’s F values combined with the high haplotype diversity and low nucleotide diversity indicate a recent population expansion with a selective sweep. The spread of *Ae. aegypti formosus* into urban areas most likely contributed to the expansion. This elevates the risk of transmission, and possibly more outbreaks, of diseases such as dengue fever. Even though *Ae. aegypti formosus* is considered a weaker vector of arboviruses, its changing habits such as moving into urban areas makes it crucial for regular entomological surveillance so as to monitor it extensively and to implement interventions aimed at controlling the spread of dengue virus in the coastal region of Kenya with the goal of preventing more outbreaks from occurring.

## Supporting information

S1 Table Lamu County mosquito collection data. (DOCX)

S1 File Number of mosquitoes sampled according to site. (PDF)

S2 File Number of mosquitoes sampled according to site and species. (PDF)
