# Supplementary material for: Vector competence for dengue-2 virus of permethrin resistant/ susceptible Aedes aegypti sub-populations from selected sites in Lamu County, Kenya
Source: PLoS One. 2026 Oct 7;21(10):e0359810. doi: 10.1371/journal.pone.0359810 (PMC13645031; doi:10.1371/journal.pone.0359810)
Supplement: Supplementary file 1 [file pone.0359810.s002.pdf]

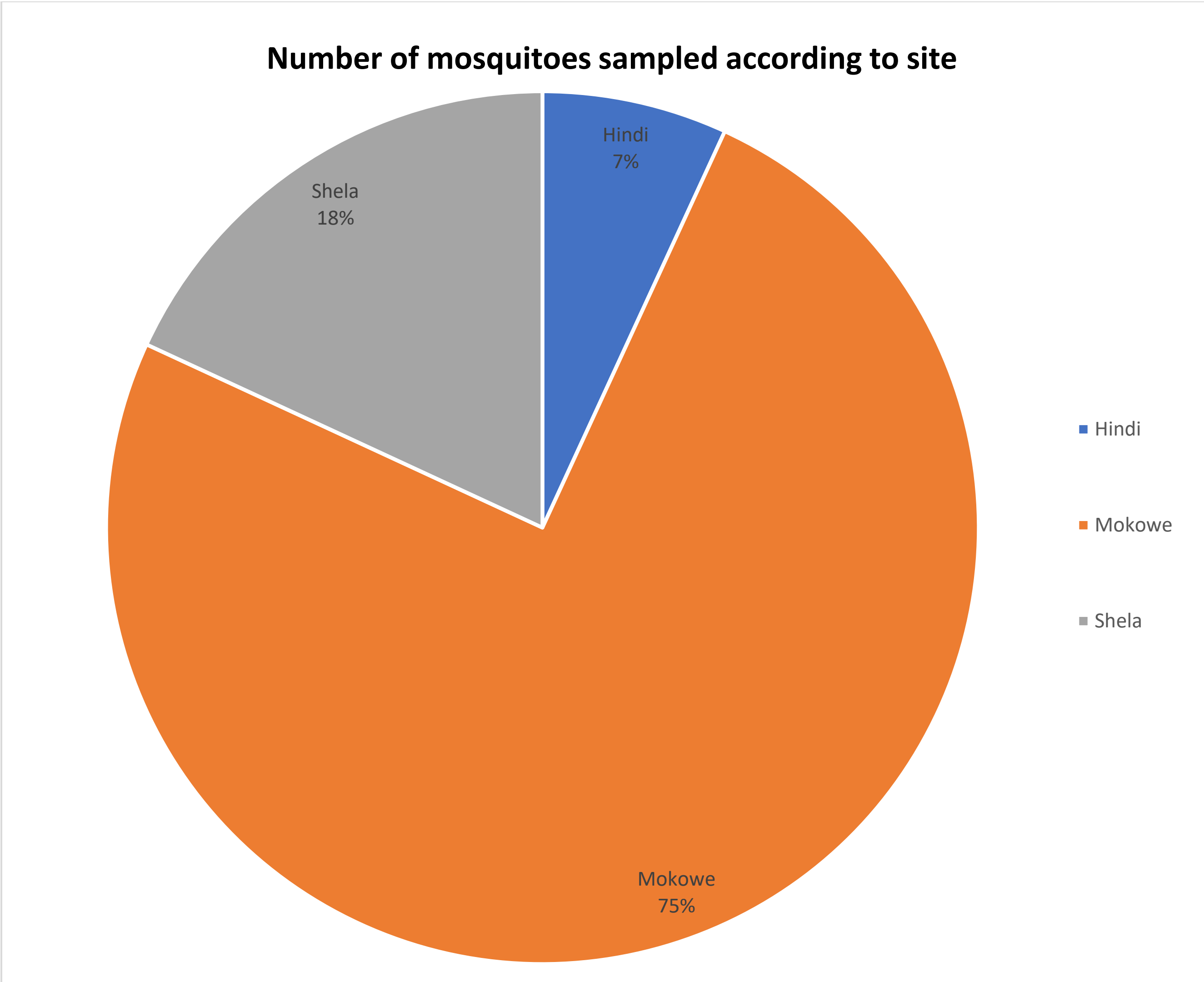


| Site | Sum of Pool Size |
|---|---|
| Hindi | 347 |
| Mokowe | 3792 |
| Shela | 915 |
| **Total** | **5054** |
