# Supplementary material for: Vector competence for dengue-2 virus of permethrin resistant/ susceptible Aedes aegypti sub-populations from selected sites in Lamu County, Kenya
Source: PLoS One. 2026 Oct 7;21(10):e0359810. doi: 10.1371/journal.pone.0359810 (PMC13645031; doi:10.1371/journal.pone.0359810)
Supplement: Supplementary file 2 [file pone.0359810.s003.pdf]

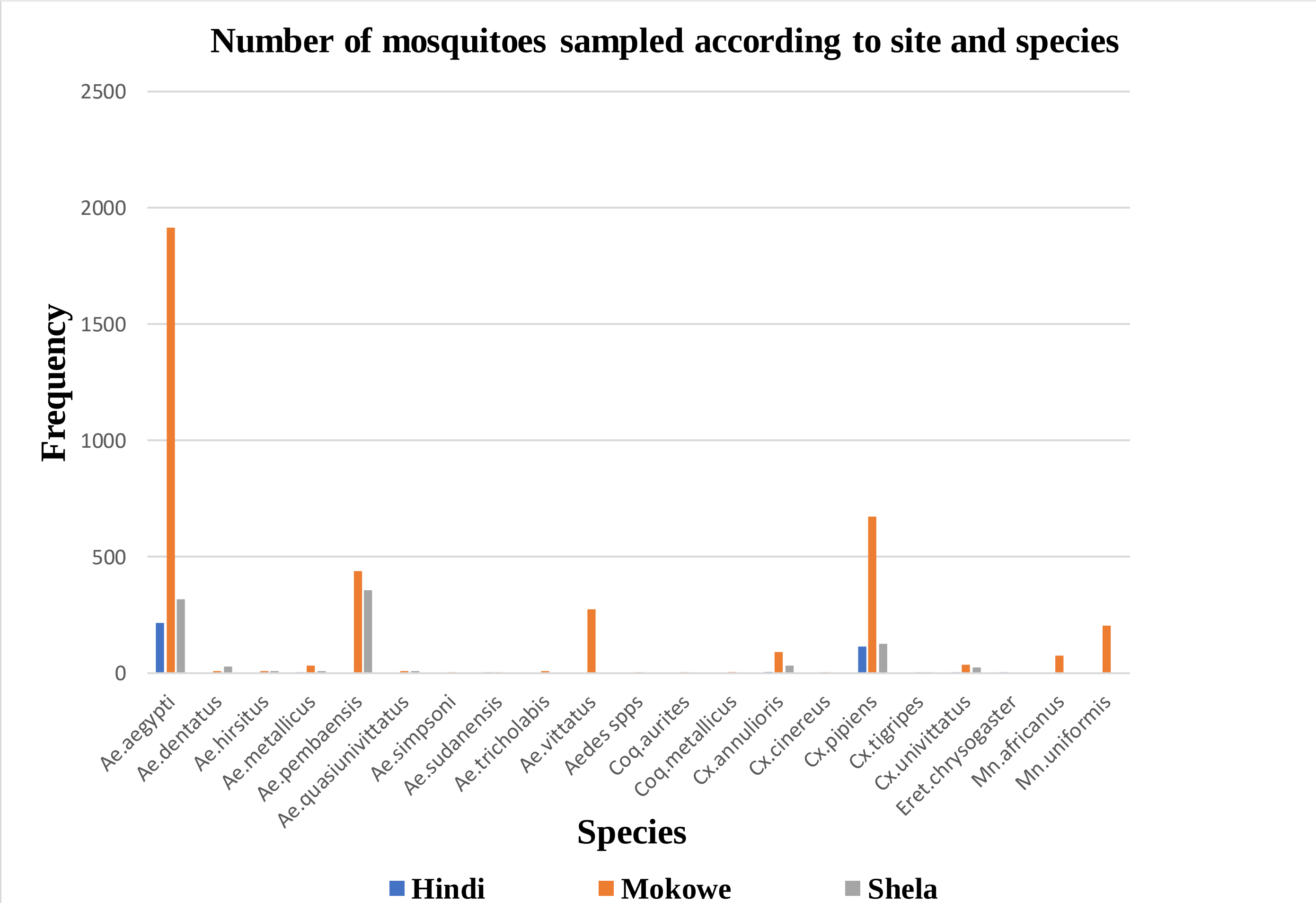

Species	Hindi	Mokowe	Shela	Grand Total
Ae.aegypti	215	1916	316	2447
Ae.dentatus		8	28	36
Ae.hirsitus		10	8	18
Ae.metallicus	3	32	10	45
Ae.pembaensis		440	357	797
Ae.quasiunivittatus		11	9	20
Ae.simpsoni		1		1
Ae.sudanensis	2	2		4
Ae.tricholabis		8		8
Ae.vittatus		275		275
Aedes spps		1		1
Coq.aurites		3		3
Coq.metallicus		5		5

Cx.annulioris	7	91	34	132
Cx.cinereus		1		1
Cx.pipiens	116	672	127	915
Cx.tigripes		1	3	4
Cx.univittatus	3	38	23	64
Eret.chrysogaster	1			1
Mn.africanus		74		74
Mn.uniformis		203		203
**Grand Total**	**347**	**3792**	**915**	**5054**
