# Supplementary material for: Vector competence for dengue-2 virus of permethrin resistant/ susceptible Aedes aegypti sub-populations from selected sites in Lamu County, Kenya
Source: PLoS One. 2026 Oct 7;21(10):e0359810. doi: 10.1371/journal.pone.0359810 (PMC13645031; doi:10.1371/journal.pone.0359810)
Supplement: Supplementary file 3 [file pone.0359810.s001.docx]

| **LAMU COUNTY MOSQUITO COLLECTION DATA** | | | | | | | | | | |
| --- | --- | --- | --- | --- | --- | --- | --- | --- | --- | --- |
| **Coll. Date** | **Area** | **Site** | **Site #** | **Trap number** | **Pool number** | **Coll. Method** | **Sex** | **Pool Size** | ***Species*** | **Box #** |
| 05-04-23 | Lamu | Shela | S4 | 4 | LAM/01 | Light Trap | F | 21 | *Cx.pipiens* | 1 |
| 05-04-23 | Lamu | Shela | S4 | 4 | LAM/02 | Light Trap | F | 1 | *Ae.pembaensis* | 1 |
| 05-04-23 | Lamu | Shela | S4 | 4 | LAM/03 | Light Trap | F | 3 | *Ae.aegypti* | 1 |
| 05-04-23 | Lamu | Shela | S4 | 4 | LAM/04 | Light Trap | M | 1 | *Ae.aegypti* | 1 |
| 05-04-23 | Lamu | Shela | S4 | 7 | LAM/05 | Light Trap | F | 4 | *Ae.aegypti* | 1 |
| 05-04-23 | Lamu | Shela | S4 | 7 | LAM/06 | Light Trap | M | 2 | *Ae.aegypti* | 1 |
| 05-04-23 | Lamu | Shela | S4 | 2 | LAM/07 | Light Trap | F | 25 | *Ae.pembaensis* | 1 |
| 05-04-23 | Lamu | Shela | S4 | 2 | LAM/08 | Light Trap | F | 13 | *Ae.pembaensis* | 1 |
| 05-04-23 | Lamu | Shela | S4 | 2 | LAM/09 | Light Trap | F | 4 | *Ae.dentatus* | 1 |
| 05-04-23 | Lamu | Shela | S4 | 3 | LAM/10 | Light Trap | F | 25 | *Ae.pembaensis* | 1 |
| 05-04-23 | Lamu | Shela | S4 | 3 | LAM/11 | Light Trap | F | 16 | *Ae.pembaensis* | 1 |
| 05-04-23 | Lamu | Shela | S4 | 3 | LAM/12 | Light Trap | F | 6 | *Ae.dentatus* | 1 |
| 05-04-23 | Lamu | Shela | S4 | 5 | LAM/13 | Light Trap | F | 17 | *Ae.pembaensis* | 1 |
| 05-04-23 | Lamu | Shela | S4 | 5 | LAM/14 | Light Trap | F | 5 | *Ae.dentatus* | 1 |
| 05-04-23 | Lamu | Shela | S4 | 6 | LAM/15 | Light Trap | M | 1 | *Ae.aegypti* | 1 |
| 05-04-23 | Lamu | Shela | S4 | 6 | LAM/16 | Light Trap | F | 3 | *Cx.pipiens* | 1 |
| 06-04-23 | Lamu | Hindi | S3 | 3 | LAM/17 | Light Trap | F | 1 | *Cx.pipiens* | 1 |
| 06-04-23 | Lamu | Hindi | S3 | 1 | LAM/18 | Light Trap | F | 1 | *Ae.aegypti* | 1 |
| 06-04-23 | Lamu | Hindi | S3 | 1 | LAM/19 | Light Trap | F | 1 | *Cx.pipiens* | 1 |
| 06-04-23 | Lamu | Mokowe | S2 | 6 | LAM/20 | Light Trap | M | 1 | *Ae.aegypti* | 1 |
| 06-04-23 | Lamu | Mokowe | S2 | 6 | LAM/21 | Light Trap | F | 9 | *Ae.aegypti* | 1 |
| 06-04-23 | Lamu | Mokowe | S2 | 6 | LAM/22 | Light Trap | F | 5 | *Ae.pembaensis* | 1 |
| 06-04-23 | Lamu | Mokowe | S2 | 6 | LAM/23 | Light Trap | F | 3 | *Cx.pipiens* | 1 |
| 06-04-23 | Lamu | Mokowe | S2 | 1 | LAM/24 | Light Trap | F | 4 | *Ae.aegypti* | 1 |
| 06-04-23 | Lamu | Mokowe | S2 | 1 | LAM/25 | Light Trap | F | 2 | *Cx.pipiens* | 1 |
| 06-04-23 | Lamu | Mokowe | S2 | 1 | LAM/26 | Light Trap | F | 8 | *Ae.pembaensis* | 1 |
| 06-04-23 | Lamu | Mokowe | S2 | 5 | LAM/27 | Light Trap | M | 1 | *Ae.aegypti* | 1 |
| 06-04-23 | Lamu | Mokowe | S2 | 5 | LAM/28 | Light Trap | F | 4 | *Ae.aegypti* | 1 |
| 06-04-23 | Lamu | Mokowe | S2 | 5 | LAM/29 | Light Trap | F | 2 | *Ae.pembaensis* | 1 |
| 06-04-23 | Lamu | Mokowe | S2 | 5 | LAM/30 | Light Trap | F | 3 | *Cx.pipiens* | 1 |
| 06-04-23 | Lamu | Mokowe | S2 | 4 | LAM/31 | Light Trap | M | 7 | *Ae.aegypti* | 1 |
| 06-04-23 | Lamu | Mokowe | S2 | 4 | LAM/32 | Light Trap | F | 26 | *Ae.aegypti* | 1 |
| 06-04-23 | Lamu | Mokowe | S2 | 4 | LAM/33 | Light Trap | F | 25 | *Ae.pembaensis* | 1 |
| 06-04-23 | Lamu | Mokowe | S2 | 4 | LAM/34 | Light Trap | F | 3 | *Ae.pembaensis* | 1 |
| 06-04-23 | Lamu | Mokowe | S2 | 4 | LAM/35 | Light Trap | F | 3 | *Cx.pipiens* | 1 |
| 06-04-23 | Lamu | Mokowe | S2 | 3 | LAM/36 | Light Trap | F | 1 | *Ae.aegypti* | 1 |
| 06-04-23 | Lamu | Mokowe | S2 | 3 | LAM/37 | Light Trap | F | 5 | *Ae.pembaensis* | 1 |
| 06-04-23 | Lamu | Mokowe | S2 | 3 | LAM/38 | Light Trap | F | 1 | *Cx.pipiens* | 1 |
| 06-04-23 | Lamu | Mokowe | S2 | 7 | LAM/39 | Light Trap | M | 9 | *Ae.aegypti* | 1 |
| 06-04-23 | Lamu | Mokowe | S2 | 7 | LAM/40 | Light Trap | F | 13 | *Ae.aegypti* | 1 |
| 06-04-23 | Lamu | Mokowe | S2 | 7 | LAM/41 | Light Trap | F | 7 | *Ae.pembaensis* | 1 |
| 06-04-23 | Lamu | Mokowe | S2 | 7 | LAM/42 | Light Trap | F | 4 | *Cx.pipiens* | 1 |
| 06-04-23 | Lamu | Mokowe | S2 | 2 | LAM/43 | Light Trap | M | 5 | *Ae.aegypti* | 1 |
| 06-04-23 | Lamu | Mokowe | S2 | 2 | LAM/44 | Light Trap | F | 12 | *Ae.aegypti* | 1 |
| 06-04-23 | Lamu | Mokowe | S2 | 2 | LAM/45 | Light Trap | F | 6 | *Ae.pembaensis* | 1 |
| 06-04-23 | Lamu | Mokowe | S2 | 2 | LAM/46 | Light Trap | F | 1 | *Cx.pipiens* | 1 |
| 08-04-23 | Lamu | Mokowe | S2 | 2 | LAM/47 | BG Sentinel Trap | F | 25 | *Ae.aegypti* | 1 |
| 08-04-23 | Lamu | Mokowe | S2 | 2 | LAM/48 | BG Sentinel Trap | F | 23 | *Ae.aegypti* | 1 |
| 08-04-23 | Lamu | Mokowe | S2 | 2 | LAM/49 | BG Sentinel Trap | M | 25 | *Ae.aegypti* | 1 |
| 08-04-23 | Lamu | Mokowe | S2 | 2 | LAM/50 | BG Sentinel Trap | M | 25 | *Ae.aegypti* | 1 |
| 08-04-23 | Lamu | Mokowe | S2 | 2 | LAM/51 | BG Sentinel Trap | M | 5 | *Ae.aegypti* | 1 |
| 08-04-23 | Lamu | Mokowe | S2 | 2 | LAM/52 | BG Sentinel Trap | F | 25 | *Ae.aegypti* | 1 |
| 08-04-23 | Lamu | Mokowe | S2 | 2 | LAM/53 | BG Sentinel Trap | F | 17 | *Ae.aegypti* | 1 |
| 08-04-23 | Lamu | Mokowe | S2 | 2 | LAM/54 | BG Sentinel Trap | M | 25 | *Ae.aegypti* | 1 |
| 08-04-23 | Lamu | Mokowe | S2 | 2 | LAM/55 | BG Sentinel Trap | M | 25 | *Ae.aegypti* | 1 |
| 08-04-23 | Lamu | Mokowe | S2 | 2 | LAM/56 | BG Sentinel Trap | M | 21 | *Ae.aegypti* | 1 |
| 08-04-23 | Lamu | Mokowe | S2 | 2 | LAM/57 | BG Sentinel Trap | F | 25 | *Ae.aegypti* | 1 |
| 08-04-23 | Lamu | Mokowe | S2 | 2 | LAM/58 | BG Sentinel Trap | F | 17 | *Ae.aegypti* | 1 |
| 08-04-23 | Lamu | Mokowe | S2 | 2 | LAM/59 | BG Sentinel Trap | M | 25 | *Ae.aegypti* | 1 |
| 08-04-23 | Lamu | Mokowe | S2 | 2 | LAM/60 | BG Sentinel Trap | M | 25 | *Ae.aegypti* | 1 |
| 08-04-23 | Lamu | Mokowe | S2 | 2 | LAM/61 | BG Sentinel Trap | M | 12 | *Ae.aegypti* | 1 |
| 08-04-23 | Lamu | Hindi | S1 | 2 | LAM/62 | BG Sentinel Trap | F | 25 | *Cx.pipiens* | 1 |
| 08-04-23 | Lamu | Hindi | S1 | 2 | LAM/63 | BG Sentinel Trap | F | 23 | *Cx.pipiens* | 1 |
| 08-04-23 | Lamu | Hindi | S3 | 2 | LAM/64 | BG Sentinel Trap | F | 10 | *Ae.aegypti* | 1 |
| 08-04-23 | Lamu | Hindi | S3 | 2 | LAM/65 | BG Sentinel Trap | M | 14 | *Ae.aegypti* | 1 |
| 09-04-23 | Lamu | Hindi | S1 | 1 | LAM/66 | BG Sentinel Trap | F | 1 | *Ae.aegypti* | 1 |
| 09-04-23 | Lamu | Hindi | S1 | 1 | LAM/67 | BG Sentinel Trap | F | 2 | *Cx.pipiens* | 1 |
| 09-04-23 | Lamu | Hindi | S1 | 1 | LAM/68 | BG Sentinel Trap | F | 2 | *Cx.pipiens* | 1 |
| 09-04-23 | Lamu | Hindi | S1 | 1 | LAM/69 | BG Sentinel Trap | F | 2 | *Ae.aegypti* | 1 |
| 15-04-23 | Lamu | Shela | S4 | 4 | LAM/70 | Light Trap | F | 1 | *Cx.annulioris* | 1 |
| 15-04-23 | Lamu | Shela | S4 | 4 | LAM/71 | Light Trap | F | 5 | *Cx.pipiens* | 1 |
| 15-04-23 | Lamu | Shela | S4 | 4 | LAM/72 | Light Trap | F | 1 | *Ae.metallicus* | 1 |
| 15-04-23 | Lamu | Shela | S4 | 4 | LAM/73 | Light Trap | F | 23 | *Ae.aegypti* | 1 |
| 15-04-23 | Lamu | Shela | S4 | 4 | LAM/74 | Light Trap | M | 7 | *Ae.aegypti* | 1 |
| 15-04-23 | Lamu | Shela | S4 | 3 | LAM/75 | Light Trap | F | 1 | *Cx.univittatus* | 1 |
| 15-04-23 | Lamu | Shela | S4 | 3 | LAM/76 | Light Trap | M | 4 | *Ae.aegypti* | 1 |
| 15-04-23 | Lamu | Shela | S4 | 3 | LAM/77 | Light Trap | F | 10 | *Ae.aegypti* | 1 |
| 15-04-23 | Lamu | Shela | S4 | 5 | LAM/78 | Light Trap | F | 1 | *Cx.pipiens* | 1 |
| 15-04-23 | Lamu | Shela | S4 | 5 | LAM/79 | Light Trap | F | 7 | *Cx.univittatus* | 1 |
| 15-04-23 | Lamu | Shela | S4 | 5 | LAM/80 | Light Trap | F | 1 | *Cx.annulioris* | 1 |
| 15-04-23 | Lamu | Shela | S4 | 5 | LAM/81 | Light Trap | F | 3 | *Ae.aegypti* | 1 |
| 15-04-23 | Lamu | Shela | S4 | 5 | LAM/82 | Light Trap | M | 5 | *Ae.aegypti* | 2 |
| 15-04-23 | Lamu | Shela | S4 | 5 | LAM/83 | Light Trap | F | 2 | *Ae.metallicus* | 2 |
| 15-04-23 | Lamu | Shela | S4 | 7 | LAM/84 | Light Trap | F | 1 | *Ae.dentatus* | 2 |
| 15-04-23 | Lamu | Shela | S4 | 7 | LAM/85 | Light Trap | F | 3 | *Ae.metallicus* | 2 |
| 15-04-23 | Lamu | Shela | S4 | 7 | LAM/86 | Light Trap | F | 3 | *Ae.aegypti* | 2 |
| 15-04-23 | Lamu | Shela | S4 | 7 | LAM/87 | Light Trap | M | 1 | *Ae.aegypti* | 2 |
| 15-04-23 | Lamu | Shela | S4 | 7 | LAM/88 | Light Trap | F | 9 | *Cx.annulioris* | 2 |
| 15-04-23 | Lamu | Shela | S4 | 7 | LAM/89 | Light Trap | F | 25 | *Ae.pembaensis* | 2 |
| 15-04-23 | Lamu | Shela | S4 | 7 | LAM/90 | Light Trap | F | 7 | *Ae.pembaensis* | 2 |
| 15-04-23 | Lamu | Shela | S4 | 2 | LAM/91 | Light Trap | F | 1 | *Cx.univittatus* | 2 |
| 15-04-23 | Lamu | Shela | S4 | 2 | LAM/92 | Light Trap | F | 5 | *Cx.pipiens* | 2 |
| 15-04-23 | Lamu | Shela | S4 | 2 | LAM/93 | Light Trap | F | 12 | *Ae.aegypti* | 2 |
| 15-04-23 | Lamu | Shela | S4 | 1 | LAM/94 | Light Trap | M | 4 | *Ae.aegypti* | 2 |
| 15-04-23 | Lamu | Shela | S4 | 1 | LAM/95 | Light Trap | F | 4 | *Ae.aegypti* | 2 |
| 15-04-23 | Lamu | Shela | S4 | 1 | LAM/96 | Light Trap | F | 7 | *Cx.pipiens* | 2 |
| 15-04-23 | Lamu | Shela | S4 | 6 | LAM/97 | Light Trap | F | 1 | *Cx.tigripes* | 2 |
| 15-04-23 | Lamu | Shela | S4 | 6 | LAM/98 | Light Trap | F | 3 | *Ae.pembaensis* | 2 |
| 15-04-23 | Lamu | Shela | S4 | 6 | LAM/99 | Light Trap | F | 3 | *Ae.metallicus* | 2 |
| 15-04-23 | Lamu | Shela | S4 | 6 | LAM/100 | Light Trap | F | 13 | *Cx.univittatus* | 2 |
| 15-04-23 | Lamu | Shela | S4 | 6 | LAM/101 | Light Trap | F | 11 | *Cx.pipiens* | 2 |
| 15-04-23 | Lamu | Shela | S4 | 6 | LAM/102 | Light Trap | F | 8 | *Cx.annulioris* | 2 |
| 15-04-23 | Lamu | Shela | S4 | 6 | LAM/103 | Light Trap | F | 25 | *Ae.aegypti* | 2 |
| 15-04-23 | Lamu | Shela | S4 | 6 | LAM/104 | Light Trap | F | 15 | *Ae.aegypti* | 2 |
| 15-04-23 | Lamu | Shela | S4 | 6 | LAM/105 | Light Trap | M | 16 | *Ae.aegypti* | 2 |
| 16-04-23 | Lamu | Mokowe | S2 | 2 | LAM/106 | Light Trap | F | 1 | *Cx.annulioris* | 2 |
| 16-04-23 | Lamu | Mokowe | S2 | 2 | LAM/107 | Light Trap | F | 4 | *Cx.pipiens* | 2 |
| 16-04-23 | Lamu | Mokowe | S2 | 2 | LAM/108 | Light Trap | F | 1 | *Ae.pembaensis* | 2 |
| 16-04-23 | Lamu | Mokowe | S2 | 2 | LAM/109 | Light Trap | F | 2 | *Ae.aegypti* | 2 |
| 16-04-23 | Lamu | Mokowe | S2 | 6 | LAM/110 | Light Trap | F | 1 | *Cx.univittatus* | 2 |
| 16-04-23 | Lamu | Mokowe | S2 | 6 | LAM/111 | Light Trap | F | 7 | *Cx.pipiens* | 2 |
| 16-04-23 | Lamu | Mokowe | S2 | 6 | LAM/112 | Light Trap | F | 2 | *Cx.annulioris* | 2 |
| 16-04-23 | Lamu | Mokowe | S2 | 6 | LAM/113 | Light Trap | F | 9 | *Ae.aegypti* | 2 |
| 16-04-23 | Lamu | Mokowe | S2 | 6 | LAM/114 | Light Trap | F | 6 | *Ae.pembaensis* | 2 |
| 16-04-23 | Lamu | Mokowe | S2 | 8 | LAM/115 | Light Trap | F | 1 | *Ae.tricholabis* | 2 |
| 16-04-23 | Lamu | Mokowe | S2 | 8 | LAM/116 | Light Trap | F | 1 | *Cx.univittatus* | 2 |
| 16-04-23 | Lamu | Mokowe | S2 | 8 | LAM/117 | Light Trap | F | 6 | *Cx.pipiens* | 2 |
| 16-04-23 | Lamu | Mokowe | S2 | 8 | LAM/118 | Light Trap | F | 8 | *Cx.annulioris* | 2 |
| 16-04-23 | Lamu | Mokowe | S2 | 8 | LAM/119 | Light Trap | F | 9 | *Ae.pembaensis* | 2 |
| 16-04-23 | Lamu | Mokowe | S2 | 8 | LAM/120 | Light Trap | F | 8 | *Ae.aegypti* | 2 |
| 16-04-23 | Lamu | Mokowe | S2 | 8 | LAM/121 | Light Trap | M | 4 | *Ae.aegypti* | 2 |
| 17-04-23 | Lamu | Hindi | S3 | 5 | LAM/122 | Light Trap | F | 5 | *Cx.pipiens* | 2 |
| 17-04-23 | Lamu | Hindi | S3 | 5 | LAM/123 | Light Trap | F | 1 | *Cx.univittatus* | 2 |
| 17-04-23 | Lamu | Hindi | S3 | 5 | LAM/124 | Light Trap | F | 4 | *Ae.aegypti* | 2 |
| 17-04-23 | Lamu | Hindi | S3 | 2 | LAM/125 | Light Trap | F | 1 | *Ae.sudanensis* | 2 |
| 17-04-23 | Lamu | Hindi | S3 | 2 | LAM/126 | Light Trap | F | 5 | *Cx.pipiens* | 2 |
| 17-04-23 | Lamu | Hindi | S3 | 2 | LAM/127 | Light Trap | F | 2 | *Ae.metallicus* | 2 |
| 17-04-23 | Lamu | Hindi | S3 | 2 | LAM/128 | Light Trap | F | 4 | *Ae.aegypti* | 2 |
| 17-04-23 | Lamu | Hindi | S3 | 2 | LAM/129 | Light Trap | M | 1 | *Ae.aegypti* | 2 |
| 17-04-23 | Lamu | Hindi | S3 | 7 | LAM/130 | Light Trap | F | 1 | *Cx.annulioris* | 2 |
| 17-04-23 | Lamu | Hindi | S3 | 7 | LAM/131 | Light Trap | F | 4 | *Cx.pipiens* | 2 |
| 17-04-23 | Lamu | Hindi | S3 | 7 | LAM/132 | Light Trap | F | 20 | *Ae.aegypti* | 2 |
| 17-04-23 | Lamu | Hindi | S3 | 7 | LAM/133 | Light Trap | M | 7 | *Ae.aegypti* | 2 |
| 17-04-23 | Lamu | Hindi | S3 | 3 | LAM/134 | Light Trap | F | 1 | *Ae.aegypti* | 2 |
| 17-04-23 | Lamu | Hindi | S3 | 3 | LAM/135 | Light Trap | F | 1 | *Ae.metallicus* | 2 |
| 17-04-23 | Lamu | Hindi | S3 | 3 | LAM/136 | Light Trap | F | 1 | *Cx.pipiens* | 2 |
| 17-04-23 | Lamu | Hindi | S3 | 1 | LAM/137 | Light Trap | F | 5 | *Cx.pipiens* | 2 |
| 17-04-23 | Lamu | Hindi | S3 | 1 | LAM/138 | Light Trap | F | 3 | *Cx.annulioris* | 2 |
| 17-04-23 | Lamu | Hindi | S3 | 1 | LAM/139 | Light Trap | F | 4 | *Ae.aegypti* | 2 |
| 17-04-23 | Lamu | Hindi | S3 | 1 | LAM/140 | Light Trap | M | 1 | *Ae.aegypti* | 2 |
| 17-04-23 | Lamu | Hindi | S3 | 6 | LAM/141 | Light Trap | F | 1 | *Eret.chrysogaster* | 2 |
| 17-04-23 | Lamu | Hindi | S3 | 6 | LAM/142 | Light Trap | F | 1 | *Ae.sudanensis* | 2 |
| 17-04-23 | Lamu | Hindi | S3 | 6 | LAM/143 | Light Trap | F | 3 | *Cx.annulioris* | 2 |
| 17-04-23 | Lamu | Hindi | S3 | 6 | LAM/144 | Light Trap | F | 11 | *Ae.aegypti* | 2 |
| 17-04-23 | Lamu | Hindi | S3 | 6 | LAM/145 | Light Trap | M | 3 | *Ae.aegypti* | 2 |
| 17-04-23 | Lamu | Hindi | S3 | 6 | LAM/146 | Light Trap | F | 1 | *Cx.univittatus* | 2 |
| 17-04-23 | Lamu | Hindi | S3 | 6 | LAM/147 | Light Trap | F | 8 | *Cx.pipiens* | 2 |
| 16-04-23 | Lamu | Mokowe | S2 | 7 | LAM/148 | Light Trap | M | 1 | *Ae.vittatus* | 2 |
| 16-04-23 | Lamu | Mokowe | S2 | 7 | LAM/149 | Light Trap | F | 10 | *Ae.vittatus* | 2 |
| 16-04-23 | Lamu | Mokowe | S2 | 7 | LAM/150 | Light Trap | F | 5 | *Cx.annulioris* | 2 |
| 16-04-23 | Lamu | Mokowe | S2 | 7 | LAM/151 | Light Trap | F | 10 | *Ae.pembaensis* | 2 |
| 16-04-23 | Lamu | Mokowe | S2 | 7 | LAM/152 | Light Trap | M | 3 | *Ae.aegypti* | 2 |
| 16-04-23 | Lamu | Mokowe | S2 | 7 | LAM/153 | Light Trap | F | 15 | *Ae.aegypti* | 2 |
| 16-04-23 | Lamu | Mokowe | S2 | 7 | LAM/154 | Light Trap | F | 25 | *Cx.pipiens* | 2 |
| 16-04-23 | Lamu | Mokowe | S2 | 7 | LAM/155 | Light Trap | F | 25 | *Cx.pipiens* | 2 |
| 16-04-23 | Lamu | Mokowe | S2 | 7 | LAM/156 | Light Trap | F | 25 | *Cx.pipiens* | 2 |
| 16-04-23 | Lamu | Mokowe | S2 | 7 | LAM/157 | Light Trap | F | 25 | *Cx.pipiens* | 2 |
| 16-04-23 | Lamu | Mokowe | S2 | 1 | LAM/158 | Light Trap | M | 6 | *Ae.aegypti* | 2 |
| 16-04-23 | Lamu | Mokowe | S2 | 1 | LAM/159 | Light Trap | F | 15 | *Ae.aegypti* | 2 |
| 16-04-23 | Lamu | Mokowe | S2 | 1 | LAM/160 | Light Trap | F | 3 | *Ae.metallicus* | 2 |
| 16-04-23 | Lamu | Mokowe | S2 | 1 | LAM/161 | Light Trap | F | 1 | *Ae.sudanensis* | 2 |
| 16-04-23 | Lamu | Mokowe | S2 | 1 | LAM/162 | Light Trap | F | 2 | *Ae.dentatus* | 2 |
| 16-04-23 | Lamu | Mokowe | S2 | 1 | LAM/163 | Light Trap | F | 8 | *Cx.univittatus* | 3 |
| 16-04-23 | Lamu | Mokowe | S2 | 1 | LAM/164 | Light Trap | F | 1 | *Aedes spps* | 3 |
| 16-04-23 | Lamu | Mokowe | S2 | 1 | LAM/165 | Light Trap | F | 14 | *Ae.pembaensis* | 3 |
| 16-04-23 | Lamu | Mokowe | S2 | 1 | LAM/166 | Light Trap | F | 19 | *Cx.annulioris* | 3 |
| 16-04-23 | Lamu | Mokowe | S2 | 1 | LAM/167 | Light Trap | F | 25 | *Cx.pipiens* | 3 |
| 16-04-23 | Lamu | Mokowe | S2 | 1 | LAM/168 | Light Trap | F | 25 | *Cx.pipiens* | 3 |
| 16-04-23 | Lamu | Mokowe | S2 | 1 | LAM/169 | Light Trap | F | 26 | *Cx.pipiens* | 3 |
| 16-04-23 | Lamu | Mokowe | S2 | 1 | LAM/170 | Light Trap | M | 23 | *Ae.vittatus* | 3 |
| 16-04-23 | Lamu | Mokowe | S2 | 1 | LAM/171 | Light Trap | F | 25 | *Ae.vittatus* | 3 |
| 16-04-23 | Lamu | Mokowe | S2 | 1 | LAM/172 | Light Trap | F | 25 | *Ae.vittatus* | 3 |
| 16-04-23 | Lamu | Mokowe | S2 | 1 | LAM/173 | Light Trap | F | 17 | *Ae.vittatus* | 3 |
| 16-04-23 | Lamu | Mokowe | S2 | 3 | LAM/174 | Light Trap | F | 2 | *Ae.tricholabis* | 3 |
| 16-04-23 | Lamu | Mokowe | S2 | 3 | LAM/175 | Light Trap | F | 1 | *Cx.univittatus* | 3 |
| 16-04-23 | Lamu | Mokowe | S2 | 3 | LAM/176 | Light Trap | F | 25 | *Ae.pembaensis* | 3 |
| 16-04-23 | Lamu | Mokowe | S2 | 3 | LAM/177 | Light Trap | F | 14 | *Ae.pembaensis* | 3 |
| 16-04-23 | Lamu | Mokowe | S2 | 3 | LAM/178 | Light Trap | F | 17 | *Cx.annulioris* | 3 |
| 16-04-23 | Lamu | Mokowe | S2 | 3 | LAM/179 | Light Trap | F | 25 | *Cx.pipiens* | 3 |
| 16-04-23 | Lamu | Mokowe | S2 | 3 | LAM/180 | Light Trap | F | 25 | *Cx.pipiens* | 3 |
| 16-04-23 | Lamu | Mokowe | S2 | 3 | LAM/181 | Light Trap | F | 20 | *Cx.pipiens* | 3 |
| 16-04-23 | Lamu | Mokowe | S2 | 3 | LAM/182 | Light Trap | M | 5 | *Ae.vittatus* | 3 |
| 16-04-23 | Lamu | Mokowe | S2 | 3 | LAM/183 | Light Trap | F | 8 | *Ae.vittatus* | 3 |
| 16-04-23 | Lamu | Mokowe | S2 | 3 | LAM/184 | Light Trap | M | 9 | *Ae.aegypti* | 3 |
| 16-04-23 | Lamu | Mokowe | S2 | 3 | LAM/185 | Light Trap | F | 25 | *Ae.aegypti* | 3 |
| 16-04-23 | Lamu | Mokowe | S2 | 3 | LAM/186 | Light Trap | F | 25 | *Ae.aegypti* | 3 |
| 16-04-23 | Lamu | Mokowe | S2 | 3 | LAM/187 | Light Trap | F | 21 | *Ae.aegypti* | 3 |
| 16-04-23 | Lamu | Mokowe | S2 | 5 | LAM/188 | Light Trap | M | 2 | *Ae.vittatus* | 3 |
| 16-04-23 | Lamu | Mokowe | S2 | 5 | LAM/189 | Light Trap | F | 6 | *Ae.vittatus* | 3 |
| 16-04-23 | Lamu | Mokowe | S2 | 5 | LAM/190 | Light Trap | M | 12 | *Ae.aegypti* | 3 |
| 16-04-23 | Lamu | Mokowe | S2 | 5 | LAM/191 | Light Trap | F | 25 | *Ae.aegypti* | 3 |
| 16-04-23 | Lamu | Mokowe | S2 | 5 | LAM/192 | Light Trap | F | 23 | *Ae.aegypti* | 3 |
| 16-04-23 | Lamu | Mokowe | S2 | 5 | LAM/193 | Light Trap | F | 25 | *Cx.pipiens* | 3 |
| 16-04-23 | Lamu | Mokowe | S2 | 5 | LAM/194 | Light Trap | F | 25 | *Cx.pipiens* | 3 |
| 16-04-23 | Lamu | Mokowe | S2 | 5 | LAM/195 | Light Trap | F | 25 | *Cx.pipiens* | 3 |
| 16-04-23 | Lamu | Mokowe | S2 | 5 | LAM/196 | Light Trap | F | 8 | *Cx.pipiens* | 3 |
| 16-04-23 | Lamu | Mokowe | S2 | 5 | LAM/197 | Light Trap | F | 25 | *Ae.pembaensis* | 3 |
| 16-04-23 | Lamu | Mokowe | S2 | 5 | LAM/198 | Light Trap | F | 21 | *Ae.pembaensis* | 3 |
| 16-04-23 | Lamu | Mokowe | S2 | 5 | LAM/199 | Light Trap | F | 16 | *Cx.annulioris* | 3 |
| 13-05-23 | Lamu | Shela | S4 | 3 | LAM/200 | Light Trap | F | 1 | *Ae.aegypti* | 1b |
| 13-05-23 | Lamu | Shela | S4 | 3 | LAM/201 | Light Trap | F | 25 | *Ae.pembaensis* | 1b |
| 13-05-23 | Lamu | Shela | S4 | 3 | LAM/202 | Light Trap | F | 2 | *Ae.pembaensis* | 1b |
| 13-05-23 | Lamu | Shela | S4 | 3 | LAM/203 | Light Trap | F | 8 | *Ae.dentatus* | 1b |
| 13-05-23 | Lamu | Shela | S4 | 3 | LAM/204 | Light Trap | F | 4 | *Cx.annulioris* | 1b |
| 13-05-23 | Lamu | Shela | S4 | 1 | LAM/205 | Light Trap | F | 1 | *Cx.pipiens* | 1b |
| 13-05-23 | Lamu | Shela | S4 | 1 | LAM/206 | Light Trap | F | 1 | *Cx.annulioris* | 1b |
| 13-05-23 | Lamu | Shela | S4 | 1 | LAM/207 | Light Trap | F | 1 | *Cx.univittatus* | 1b |
| 13-05-23 | Lamu | Shela | S4 | 1 | LAM/208 | Light Trap | F | 1 | *Ae.metallicus* | 1b |
| 13-05-23 | Lamu | Shela | S4 | 1 | LAM/209 | Light Trap | F | 2 | *Ae.dentatus* | 1b |
| 13-05-23 | Lamu | Shela | S4 | 1 | LAM/210 | Light Trap | F | 10 | *Ae.pembaensis* | 1b |
| 13-05-23 | Lamu | Shela | S4 | 2 | LAM/211 | Light Trap | F | 4 | *Ae.quasiunivittatus* | 1b |
| 13-05-23 | Lamu | Shela | S4 | 2 | LAM/212 | Light Trap | F | 10 | *Ae.pembaensis* | 1b |
| 13-05-23 | Lamu | Shela | S4 | 6 | LAM/213 | Light Trap | F | 1 | *Cx.pipiens* | 1b |
| 13-05-23 | Lamu | Shela | S4 | 6 | LAM/214 | Light Trap | F | 1 | *Ae.quasiunivittatus* | 1b |
| 13-05-23 | Lamu | Shela | S4 | 6 | LAM/215 | Light Trap | F | 5 | *Cx.annulioris* | 1b |
| 13-05-23 | Lamu | Shela | S4 | 6 | LAM/216 | Light Trap | F | 25 | *Ae.pembaensis* | 1b |
| 13-05-23 | Lamu | Shela | S4 | 6 | LAM/217 | Light Trap | F | 9 | *Ae.pembaensis* | 1b |
| 13-05-23 | Lamu | Shela | S4 | 3 | LAM/218 | Light Trap | F | 1 | *Cx.tigripes* | 1b |
| 13-05-23 | Lamu | Shela | S4 | 3 | LAM/219 | Light Trap | F | 6 | *Ae.aegypti* | 1b |
| 13-05-23 | Lamu | Shela | S4 | 3 | LAM/220 | Light Trap | F | 12 | *Cx.pipiens* | 1b |
| 10-05-23 | Lamu | Mokowe | S2 | 4 | LAM/221 | Light Trap | F | 1 | *Ae.tricholabis* | 1b |
| 10-05-23 | Lamu | Mokowe | S2 | 4 | LAM/222 | Light Trap | F | 1 | *Cx.tigripes* | 1b |
| 10-05-23 | Lamu | Mokowe | S2 | 4 | LAM/223 | Light Trap | F | 2 | *Ae.dentatus* | 1b |
| 10-05-23 | Lamu | Mokowe | S2 | 4 | LAM/224 | Light Trap | F | 1 | *Ae.vittatus* | 1b |
| 10-05-23 | Lamu | Mokowe | S2 | 4 | LAM/225 | Light Trap | M | 2 | *Ae.aegypti* | 1b |
| 10-05-23 | Lamu | Mokowe | S2 | 4 | LAM/226 | Light Trap | F | 10 | *Ae.aegypti* | 1b |
| 10-05-23 | Lamu | Mokowe | S2 | 4 | LAM/227 | Light Trap | F | 10 | *Ae.pembaensis* | 1b |
| 10-05-23 | Lamu | Mokowe | S2 | 4 | LAM/228 | Light Trap | F | 23 | *Cx.pipiens* | 1b |
| 05-05-23 | Lamu | Shela | S4 | 2 | LAM/229 | Light Trap | M | 1 | *Ae.aegypti* | 1b |
| 05-05-23 | Lamu | Shela | S4 | 2 | LAM/230 | Light Trap | F | 7 | *Ae.aegypti* | 1b |
| 05-05-23 | Lamu | Shela | S4 | 2 | LAM/231 | Light Trap | F | 17 | *Cx.pipiens* | 1b |
| 13-05-23 | Lamu | Shela | S4 | 4 | LAM/232 | Light Trap | F | 1 | *Ae.dentatus* | 1b |
| 13-05-23 | Lamu | Shela | S4 | 4 | LAM/233 | Light Trap | F | 4 | *Cx.annulioris* | 1b |
| 13-05-23 | Lamu | Shela | S4 | 4 | LAM/234 | Light Trap | F | 2 | *Ae.aegypti* | 1b |
| 13-05-23 | Lamu | Shela | S4 | 4 | LAM/235 | Light Trap | F | 25 | *Ae.pembaensis* | 1b |
| 13-05-23 | Lamu | Shela | S4 | 4 | LAM/236 | Light Trap | F | 18 | *Ae.pembaensis* | 1b |
| 05-05-23 | Lamu | Shela | S4 | 6 | LAM/237 | Light Trap | F | 1 | *Cx.tigripes* | 1b |
| 05-05-23 | Lamu | Shela | S4 | 6 | LAM/238 | Light Trap | F | 1 | *Cx.annulioris* | 1b |
| 05-05-23 | Lamu | Shela | S4 | 6 | LAM/239 | Light Trap | F | 7 | *Cx.pipiens* | 1b |
| 11-05-23 | Lamu | Mokowe | S2 | 6 | LAM/230 | Light Trap | F | 1 | *Ae.dentatus* | 1b |
| 11-05-23 | Lamu | Mokowe | S2 | 6 | LAM/231 | Light Trap | F | 1 | *Ae.metallicus* | 1b |
| 11-05-23 | Lamu | Mokowe | S2 | 6 | LAM/232 | Light Trap | M | 8 | *Ae.aegypti* | 1b |
| 11-05-23 | Lamu | Mokowe | S2 | 6 | LAM/233 | Light Trap | F | 25 | *Ae.aegypti* | 1b |
| 11-05-23 | Lamu | Mokowe | S2 | 6 | LAM/234 | Light Trap | F | 25 | *Ae.aegypti* | 1b |
| 11-05-23 | Lamu | Mokowe | S2 | 6 | LAM/235 | Light Trap | F | 16 | *Ae.aegypti* | 1b |
| 11-05-23 | Lamu | Mokowe | S2 | 6 | LAM/236 | Light Trap | F | 4 | *Cx.annulioris* | 1b |
| 11-05-23 | Lamu | Mokowe | S2 | 6 | LAM/237 | Light Trap | F | 6 | *Cx.pipiens* | 1b |
| 11-05-23 | Lamu | Mokowe | S2 | 6 | LAM/238 | Light Trap | F | 20 | *Ae.pembaensis* | 1b |
| 10-05-23 | Lamu | Mokowe | S2 | 1 | LAM/239 | Light Trap | F | 4 | *Ae.vittatus* | 1b |
| 10-05-23 | Lamu | Mokowe | S2 | 1 | LAM/250 | Light Trap | F | 1 | *Ae.metallicus* | 1b |
| 10-05-23 | Lamu | Mokowe | S2 | 1 | LAM/251 | Light Trap | F | 4 | *Cx.annulioris* | 1b |
| 10-05-23 | Lamu | Mokowe | S2 | 1 | LAM/252 | Light Trap | M | 14 | *Ae.aegypti* | 1b |
| 10-05-23 | Lamu | Mokowe | S2 | 1 | LAM/253 | Light Trap | F | 25 | *Ae.aegypti* | 1b |
| 10-05-23 | Lamu | Mokowe | S2 | 1 | LAM/254 | Light Trap | F | 25 | *Ae.aegypti* | 1b |
| 10-05-23 | Lamu | Mokowe | S2 | 1 | LAM/255 | Light Trap | F | 6 | *Ae.aegypti* | 1b |
| 10-05-23 | Lamu | Mokowe | S2 | 1 | LAM/256 | Light Trap | F | 25 | *Cx.pipiens* | 1b |
| 10-05-23 | Lamu | Mokowe | S2 | 1 | LAM/257 | Light Trap | F | 7 | *Cx.pipiens* | 1b |
| 10-05-23 | Lamu | Mokowe | S2 | 1 | LAM/258 | Light Trap | F | 25 | *Ae.pembaensis* | 1b |
| 10-05-23 | Lamu | Mokowe | S2 | 1 | LAM/259 | Light Trap | F | 5 | *Ae.pembaensis* | 1b |
| 11-05-23 | Lamu | Mokowe | S2 | 2 | LAM/260 | Light Trap | F | 2 | *Ae.metallicus* | 1b |
| 11-05-23 | Lamu | Mokowe | S2 | 2 | LAM/261 | Light Trap | F | 4 | *Cx.annulioris* | 1b |
| 11-05-23 | Lamu | Mokowe | S2 | 2 | LAM/262 | Light Trap | F | 3 | *Ae.dentatus* | 1b |
| 11-05-23 | Lamu | Mokowe | S2 | 2 | LAM/263 | Light Trap | F | 6 | *Cx.pipiens* | 1b |
| 11-05-23 | Lamu | Mokowe | S2 | 2 | LAM/264 | Light Trap | M | 17 | *Ae.aegypti* | 1b |
| 11-05-23 | Lamu | Mokowe | S2 | 2 | LAM/265 | Light Trap | F | 25 | *Ae.aegypti* | 1b |
| 11-05-23 | Lamu | Mokowe | S2 | 2 | LAM/266 | Light Trap | F | 25 | *Ae.aegypti* | 1b |
| 11-05-23 | Lamu | Mokowe | S2 | 2 | LAM/267 | Light Trap | F | 11 | *Ae.aegypti* | 1b |
| 11-05-23 | Lamu | Mokowe | S2 | 2 | LAM/268 | Light Trap | F | 25 | *Ae.pembaensis* | 1b |
| 11-05-23 | Lamu | Mokowe | S2 | 2 | LAM/269 | Light Trap | F | 5 | *Ae.pembaensis* | 1b |
| 11-05-23 | Lamu | Mokowe | S2 | 5 | LAM/270 | Light Trap | F | 10 | *Cx.univittatus* | 1b |
| 11-05-23 | Lamu | Mokowe | S2 | 5 | LAM/271 | Light Trap | F | 1 | *Cx.cinereus* | 1b |
| 11-05-23 | Lamu | Mokowe | S2 | 5 | LAM/272 | Light Trap | F | 2 | *Ae.metallicus* | 1b |
| 11-05-23 | Lamu | Mokowe | S2 | 5 | LAM/273 | Light Trap | M | 5 | *Ae.aegypti* | 1b |
| 11-05-23 | Lamu | Mokowe | S2 | 5 | LAM/274 | Light Trap | F | 25 | *Ae.aegypti* | 1b |
| 11-05-23 | Lamu | Mokowe | S2 | 5 | LAM/275 | Light Trap | F | 6 | *Ae.aegypti* | 1b |
| 11-05-23 | Lamu | Mokowe | S2 | 5 | LAM/276 | Light Trap | F | 20 | *Cx.pipiens* | 1b |
| 11-05-23 | Lamu | Mokowe | S2 | 5 | LAM/277 | Light Trap | F | 12 | *Ae.pembaensis* | 1b |
| 05-05-23 | Lamu | Mokowe | S2 | 4 | LAM/278 | Light Trap | F | 11 | *Cx.pipiens* | 1b |
| 05-05-23 | Lamu | Mokowe | S2 | 4 | LAM/279 | Light Trap | F | 1 | *Ae.aegypti* | 1b |
| 05-05-23 | Lamu | Mokowe | S2 | 5 | LAM/280 | Light Trap | F | 2 | *Ae.metallicus* | 1b |
| 05-05-23 | Lamu | Mokowe | S2 | 5 | LAM/281 | Light Trap | F | 5 | *Ae.aegypti* | 2b |
| 05-05-23 | Lamu | Mokowe | S2 | 5 | LAM/282 | Light Trap | F | 12 | *Cx.pipiens* | 2b |
| 10-05-23 | Lamu | Mokowe | S2 | 2 | LAM/283 | Light Trap | F | 1 | *Ae.vittatus* | 2b |
| 10-05-23 | Lamu | Mokowe | S2 | 2 | LAM/284 | Light Trap | F | 2 | *Ae.metallicus* | 2b |
| 10-05-23 | Lamu | Mokowe | S2 | 2 | LAM/285 | Light Trap | F | 18 | *Cx.pipiens* | 2b |
| 10-05-23 | Lamu | Mokowe | S2 | 2 | LAM/286 | Light Trap | M | 12 | *Ae.aegypti* | 2b |
| 10-05-23 | Lamu | Mokowe | S2 | 2 | LAM/287 | Light Trap | F | 23 | *Ae.aegypti* | 2b |
| 10-05-23 | Lamu | Mokowe | S2 | 2 | LAM/288 | Light Trap | F | 5 | *Ae.pembaensis* | 2b |
| 11-05-23 | Lamu | Mokowe | S2 | 1 | LAM/289 | Light Trap | F | 2 | *Cx.annulioris* | 2b |
| 11-05-23 | Lamu | Mokowe | S2 | 1 | LAM/290 | Light Trap | F | 1 | *Ae.metallicus* | 2b |
| 11-05-23 | Lamu | Mokowe | S2 | 1 | LAM/291 | Light Trap | F | 2 | *Cx.pipiens* | 2b |
| 11-05-23 | Lamu | Mokowe | S2 | 1 | LAM/292 | Light Trap | F | 5 | *Ae.quasiunivittatus* | 2b |
| 11-05-23 | Lamu | Mokowe | S2 | 1 | LAM/293 | Light Trap | F | 4 | *Cx.univittatus* | 2b |
| 11-05-23 | Lamu | Mokowe | S2 | 1 | LAM/294 | Light Trap | M | 6 | *Ae.aegypti* | 2b |
| 11-05-23 | Lamu | Mokowe | S2 | 1 | LAM/295 | Light Trap | F | 25 | *Ae.aegypti* | 2b |
| 11-05-23 | Lamu | Mokowe | S2 | 1 | LAM/296 | Light Trap | F | 21 | *Ae.pembaensis* | 2b |
| 11-05-23 | Lamu | Mokowe | S2 | 1 | LAM/297 | Light Trap | F | 25 | *Ae.aegypti* | 2b |
| 11-05-23 | Lamu | Mokowe | S2 | 1 | LAM/298 | Light Trap | F | 6 | *Ae.aegypti* | 2b |
| 11-05-23 | Lamu | Mokowe | S2 | 3 | LAM/299 | Light Trap | F | 1 | *Ae.vittatus* | 2b |
| 11-05-23 | Lamu | Mokowe | S2 | 3 | LAM/300 | Light Trap | F | 8 | *Cx.univittatus* | 2b |
| 11-05-23 | Lamu | Mokowe | S2 | 3 | LAM/301 | Light Trap | F | 2 | *Cx.annulioris* | 2b |
| 11-05-23 | Lamu | Mokowe | S2 | 3 | LAM/302 | Light Trap | F | 1 | *Ae.metallicus* | 2b |
| 11-05-23 | Lamu | Mokowe | S2 | 3 | LAM/303 | Light Trap | F | 6 | *Cx.pipiens* | 2b |
| 11-05-23 | Lamu | Mokowe | S2 | 3 | LAM/304 | Light Trap | M | 11 | *Ae.aegypti* | 2b |
| 11-05-23 | Lamu | Mokowe | S2 | 3 | LAM/305 | Light Trap | F | 25 | *Ae.aegypti* | 2b |
| 11-05-23 | Lamu | Mokowe | S2 | 3 | LAM/306 | Light Trap | F | 25 | *Ae.aegypti* | 2b |
| 11-05-23 | Lamu | Mokowe | S2 | 3 | LAM/307 | Light Trap | F | 25 | *Ae.aegypti* | 2b |
| 11-05-23 | Lamu | Mokowe | S2 | 3 | LAM/308 | Light Trap | F | 25 | *Ae.aegypti* | 2b |
| 11-05-23 | Lamu | Mokowe | S2 | 3 | LAM/309 | Light Trap | F | 13 | *Ae.aegypti* | 2b |
| 11-05-23 | Lamu | Mokowe | S2 | 3 | LAM/310 | Light Trap | F | 25 | *Ae.pembaensis* | 2b |
| 11-05-23 | Lamu | Mokowe | S2 | 3 | LAM/311 | Light Trap | F | 12 | *Ae.pembaensis* | 2b |
| 10-05-23 | Lamu | Mokowe | S2 | 3 | LAM/312 | Light Trap | F | 1 | *Cx.annulioris* | 2b |
| 10-05-23 | Lamu | Mokowe | S2 | 3 | LAM/313 | Light Trap | F | 2 | *Ae.quasiunivittatus* | 2b |
| 10-05-23 | Lamu | Mokowe | S2 | 3 | LAM/314 | Light Trap | F | 6 | *Ae.pembaensis* | 2b |
| 10-05-23 | Lamu | Mokowe | S2 | 3 | LAM/315 | Light Trap | M | 13 | *Ae.aegypti* | 2b |
| 10-05-23 | Lamu | Mokowe | S2 | 3 | LAM/316 | Light Trap | F | 14 | *Ae.aegypti* | 2b |
| 10-05-23 | Lamu | Mokowe | S2 | 3 | LAM/317 | Light Trap | F | 25 | *Cx.pipiens* | 2b |
| 10-05-23 | Lamu | Mokowe | S2 | 3 | LAM/318 | Light Trap | F | 25 | *Cx.pipiens* | 2b |
| 10-05-23 | Lamu | Mokowe | S2 | 3 | LAM/319 | Light Trap | F | 25 | *Cx.pipiens* | 2b |
| 10-05-23 | Lamu | Mokowe | S2 | 3 | LAM/320 | Light Trap | F | 5 | *Cx.pipiens* | 2b |
| 13-05-23 | Lamu | Shela | S4 | 5 | LAM/321 | Light Trap | F | 1 | *Ae.dentatus* | 2b |
| 13-05-23 | Lamu | Shela | S4 | 5 | LAM/323 | Light Trap | F | 1 | *Cx.pipiens* | 2b |
| 13-05-23 | Lamu | Shela | S4 | 5 | LAM/323 | Light Trap | F | 25 | *Ae.pembaensis* | 2b |
| 13-05-23 | Lamu | Shela | S4 | 5 | LAM/323 | Light Trap | F | 25 | *Ae.pembaensis* | 2b |
| 13-05-23 | Lamu | Shela | S4 | 5 | LAM/325 | Light Trap | F | 23 | *Ae.pembaensis* | 2b |
| 10-05-23 | Lamu | Mokowe | S2 | 5 | LAM/326 | Light Trap | F | 1 | *Cx.annulioris* | 2b |
| 10-05-23 | Lamu | Mokowe | S2 | 5 | LAM/327 | Light Trap | F | 5 | *Ae.pembaensis* | 2b |
| 10-05-23 | Lamu | Mokowe | S2 | 5 | LAM/328 | Light Trap | M | 1 | *Ae.aegypti* | 2b |
| 10-05-23 | Lamu | Mokowe | S2 | 5 | LAM/329 | Light Trap | F | 25 | *Ae.aegypti* | 2b |
| 10-05-23 | Lamu | Mokowe | S2 | 5 | LAM/330 | Light Trap | F | 3 | *Ae.aegypti* | 2b |
| 10-05-23 | Lamu | Mokowe | S2 | 5 | LAM/331 | Light Trap | F | 25 | *Cx.pipiens* | 2b |
| 10-05-23 | Lamu | Mokowe | S2 | 5 | LAM/332 | Light Trap | F | 15 | *Cx.pipiens* | 2b |
| 10-05-23 | Lamu | Mokowe | S2 | 6 | LAM/333 | Light Trap | F | 1 | *Cx.annulioris* | 2b |
| 10-05-23 | Lamu | Mokowe | S2 | 6 | LAM/334 | Light Trap | M | 2 | *Ae.aegypti* | 2b |
| 10-05-23 | Lamu | Mokowe | S2 | 6 | LAM/335 | Light Trap | F | 10 | *Ae.pembaensis* | 2b |
| 10-05-23 | Lamu | Mokowe | S2 | 6 | LAM/336 | Light Trap | F | 11 | *Cx.pipiens* | 2b |
| 10-05-23 | Lamu | Mokowe | S2 | 6 | LAM/337 | Light Trap | F | 25 | *Ae.aegypti* | 2b |
| 10-05-23 | Lamu | Mokowe | S2 | 6 | LAM/338 | Light Trap | F | 23 | *Ae.aegypti* | 2b |
| 05-05-23 | Lamu | Mokowe | S2 | 1 | LAM/339 | Light Trap | F | 8 | *Ae.metallicus* | 2b |
| 05-05-23 | Lamu | Mokowe | S2 | 1 | LAM/340 | Light Trap | F | 4 | *Ae.pembaensis* | 2b |
| 05-05-23 | Lamu | Mokowe | S2 | 1 | LAM/341 | Light Trap | F | 14 | *Ae.aegypti* | 2b |
| 05-05-23 | Lamu | Mokowe | S2 | 1 | LAM/342 | Light Trap | F | 14 | *Cx.pipiens* | 2b |
| 11-05-23 | Lamu | Mokowe | S2 | 4 | LAM/343 | Light Trap | F | 1 | *Ae.vittatus* | 2b |
| 11-05-23 | Lamu | Mokowe | S2 | 4 | LAM/344 | Light Trap | F | 1 | *Cx.annulioris* | 2b |
| 11-05-23 | Lamu | Mokowe | S2 | 4 | LAM/345 | Light Trap | F | 3 | *Ae.quasiunivittatus* | 2b |
| 11-05-23 | Lamu | Mokowe | S2 | 4 | LAM/346 | Light Trap | F | 4 | *Ae.metallicus* | 2b |
| 11-05-23 | Lamu | Mokowe | S2 | 4 | LAM/347 | Light Trap | M | 4 | *Ae.aegypti* | 2b |
| 11-05-23 | Lamu | Mokowe | S2 | 4 | LAM/348 | Light Trap | F | 12 | *Ae.aegypti* | 2b |
| 11-05-23 | Lamu | Mokowe | S2 | 4 | LAM/349 | Light Trap | F | 9 | *Ae.pembaensis* | 2b |
| 02-06-23 | Lamu | Shela | S5 | 2 | LAM/350 | BG Sentinel Trap | M | 25 | *Ae.aegypti* | 2b |
| 02-06-23 | Lamu | Shela | S5 | 2 | LAM/351 | BG Sentinel Trap | M | 25 | *Ae.aegypti* | 2b |
| 02-06-23 | Lamu | Shela | S5 | 2 | LAM/352 | BG Sentinel Trap | M | 11 | *Ae.aegypti* | 2b |
| 02-06-23 | Lamu | Shela | S5 | 2 | LAM/353 | BG Sentinel Trap | F | 25 | *Ae.aegypti* | 2b |
| 02-06-23 | Lamu | Shela | S5 | 2 | LAM/354 | BG Sentinel Trap | F | 25 | *Ae.aegypti* | 2b |
| 02-06-23 | Lamu | Shela | S5 | 2 | LAM/355 | BG Sentinel Trap | F | 25 | *Ae.aegypti* | 2b |
| 02-06-23 | Lamu | Shela | S5 | 2 | LAM/356 | BG Sentinel Trap | F | 19 | *Ae.aegypti* | 2b |
| 30-06-23 | Lamu | Mokowe | S2 | 2 | LAM/357 | BG Sentinel Trap | M | 25 | *Ae.aegypti* | 2b |
| 30-06-23 | Lamu | Mokowe | S2 | 2 | LAM/358 | BG Sentinel Trap | M | 25 | *Ae.aegypti* | 2b |
| 30-06-23 | Lamu | Mokowe | S2 | 2 | LAM/359 | BG Sentinel Trap | M | 25 | *Ae.aegypti* | 2b |
| 30-06-23 | Lamu | Mokowe | S2 | 2 | LAM/360 | BG Sentinel Trap | M | 23 | *Ae.aegypti* | 2b |
| 30-06-23 | Lamu | Mokowe | S2 | 2 | LAM/361 | BG Sentinel Trap | F | 25 | *Ae.aegypti* | 2b |
| 30-06-23 | Lamu | Mokowe | S2 | 2 | LAM/362 | BG Sentinel Trap | F | 25 | *Ae.aegypti* | 3b |
| 30-06-23 | Lamu | Mokowe | S2 | 2 | LAM/363 | BG Sentinel Trap | F | 25 | *Ae.aegypti* | 3b |
| 30-06-23 | Lamu | Mokowe | S2 | 2 | LAM/364 | BG Sentinel Trap | F | 25 | *Ae.aegypti* | 3b |
| 30-06-23 | Lamu | Mokowe | S2 | 2 | LAM/365 | BG Sentinel Trap | F | 25 | *Ae.aegypti* | 3b |
| 30-06-23 | Lamu | Mokowe | S2 | 2 | LAM/366 | BG Sentinel Trap | F | 25 | *Ae.aegypti* | 3b |
| 30-06-23 | Lamu | Mokowe | S2 | 2 | LAM/367 | BG Sentinel Trap | F | 13 | *Ae.aegypti* | 3b |
| 01-06-23 | Lamu | Mokowe | S2 | 2 | LAM/368 | BG Sentinel Trap | M | 19 | *Ae.aegypti* | 3b |
| 01-06-23 | Lamu | Mokowe | S2 | 2 | LAM/369 | BG Sentinel Trap | F | 19 | *Ae.aegypti* | 3b |
| 30-06-23 | Lamu | Hindi | S3 | 2 | LAM/370 | BG Sentinel Trap | F | 1 | *Cx.univittatus* | 3b |
| 30-06-23 | Lamu | Hindi | S3 | 2 | LAM/371 | BG Sentinel Trap | F | 25 | *Cx.pipiens* | 3b |
| 30-06-23 | Lamu | Hindi | S3 | 2 | LAM/372 | BG Sentinel Trap | F | 9 | *Cx.pipiens* | 3b |
| 30-06-23 | Lamu | Hindi | S3 | 2 | LAM/373 | BG Sentinel Trap | M | 25 | *Ae.aegypti* | 3b |
| 30-06-23 | Lamu | Hindi | S3 | 2 | LAM/374 | BG Sentinel Trap | M | 25 | *Ae.aegypti* | 3b |
| 30-06-23 | Lamu | Hindi | S3 | 2 | LAM/375 | BG Sentinel Trap | M | 23 | *Ae.aegypti* | 3b |
| 30-06-23 | Lamu | Hindi | S3 | 2 | LAM/376 | BG Sentinel Trap | F | 25 | *Ae.aegypti* | 3b |
| 30-06-23 | Lamu | Hindi | S3 | 2 | LAM/377 | BG Sentinel Trap | F | 25 | *Ae.aegypti* | 3b |
| 30-06-23 | Lamu | Hindi | S3 | 2 | LAM/378 | BG Sentinel Trap | F | 8 | *Ae.aegypti* | 3b |
| 30-06-23 | Lamu | Mokowe | S2 | 6 | LAM/379 | Light Trap | F | 1 | *Ae.metallicus* | 3b |
| 30-06-23 | Lamu | Mokowe | S2 | 6 | LAM/380 | Light Trap | F | 3 | *Ae.hirsitus* | 3b |
| 30-06-23 | Lamu | Mokowe | S2 | 6 | LAM/381 | Light Trap | F | 7 | *Ae.pembaensis* | 3b |
| 30-06-23 | Lamu | Mokowe | S2 | 6 | LAM/382 | Light Trap | M | 19 | *Ae.vittatus* | 3b |
| 30-06-23 | Lamu | Mokowe | S2 | 6 | LAM/383 | Light Trap | F | 21 | *Ae.vittatus* | 3b |
| 30-06-23 | Lamu | Mokowe | S2 | 6 | LAM/384 | Light Trap | M | 7 | *Ae.aegypti* | 3b |
| 30-06-23 | Lamu | Mokowe | S2 | 6 | LAM/385 | Light Trap | F | 7 | *Ae.aegypti* | 3b |
| 30-06-23 | Lamu | Mokowe | S2 | 1 | LAM/386 | Light Trap | F | 1 | *Ae.hirsitus* | 3b |
| 30-06-23 | Lamu | Mokowe | S2 | 1 | LAM/387 | Light Trap | F | 1 | *Ae.quasiunivittatus* | 3b |
| 30-06-23 | Lamu | Mokowe | S2 | 1 | LAM/388 | Light Trap | F | 2 | *Cx.pipiens* | 3b |
| 30-06-23 | Lamu | Mokowe | S2 | 1 | LAM/389 | Light Trap | F | 3 | *Cx.univittatus* | 3b |
| 30-06-23 | Lamu | Mokowe | S2 | 1 | LAM/390 | Light Trap | F | 1 | *Ae.sudanensis* | 3b |
| 30-06-23 | Lamu | Mokowe | S2 | 1 | LAM/391 | Light Trap | M | 13 | *Ae.vittatus* | 3b |
| 30-06-23 | Lamu | Mokowe | S2 | 1 | LAM/392 | Light Trap | F | 13 | *Ae.vittatus* | 3b |
| 30-06-23 | Lamu | Mokowe | S2 | 1 | LAM/393 | Light Trap | F | 14 | *Ae.pembaensis* | 3b |
| 30-06-23 | Lamu | Mokowe | S2 | 1 | LAM/394 | Light Trap | M | 25 | *Ae.aegypti* | 3b |
| 30-06-23 | Lamu | Mokowe | S2 | 1 | LAM/395 | Light Trap | M | 14 | *Ae.aegypti* | 3b |
| 30-06-23 | Lamu | Mokowe | S2 | 1 | LAM/396 | Light Trap | F | 25 | *Ae.aegypti* | 3b |
| 30-06-23 | Lamu | Mokowe | S2 | 1 | LAM/397 | Light Trap | F | 16 | *Ae.aegypti* | 3b |
| 30-06-23 | Lamu | Mokowe | S2 | 5 | LAM/398 | Light Trap | F | 5 | *Ae.pembaensis* | 3b |
| 30-06-23 | Lamu | Mokowe | S2 | 5 | LAM/399 | Light Trap | F | 3 | *Ae.hirsitus* | 3b |
| 30-06-23 | Lamu | Mokowe | S2 | 5 | LAM/400 | Light Trap | M | 6 | *Ae.vittatus* | 3b |
| 30-06-23 | Lamu | Mokowe | S2 | 5 | LAM/401 | Light Trap | F | 14 | *Ae.vittatus* | 3b |
| 30-06-23 | Lamu | Mokowe | S2 | 5 | LAM/402 | Light Trap | M | 19 | *Ae.aegypti* | 3b |
| 30-06-23 | Lamu | Mokowe | S2 | 5 | LAM/403 | Light Trap | F | 16 | *Ae.aegypti* | 3b |
| 30-06-23 | Lamu | Mokowe | S2 | 4 | LAM/406 | Light Trap | M | 9 | *Ae.vittatus* | 3b |
| 30-06-23 | Lamu | Mokowe | S2 | 4 | LAM/405 | Light Trap | F | 9 | *Ae.vittatus* | 3b |
| 30-06-23 | Lamu | Mokowe | S2 | 4 | LAM/406 | Light Trap | F | 1 | *Cx.pipiens* | 3b |
| 30-06-23 | Lamu | Mokowe | S2 | 4 | LAM/407 | Light Trap | F | 7 | *Ae.pembaensis* | 3b |
| 30-06-23 | Lamu | Mokowe | S2 | 4 | LAM/408 | Light Trap | F | 1 | *Ae.hirsitus* | 3b |
| 30-06-23 | Lamu | Mokowe | S2 | 4 | LAM/409 | Light Trap | F | 6 | *Ae.aegypti* | 3b |
| 30-06-23 | Lamu | Mokowe | S2 | 4 | LAM/410 | Light Trap | M | 4 | *Ae.aegypti* | 3b |
| 30-06-23 | Lamu | Mokowe | S2 | 4 | LAM/411 | Light Trap | M | 3 | *Ae.vittatus* | 3b |
| 30-06-23 | Lamu | Mokowe | S2 | 3 | LAM/412 | Light Trap | F | 1 | *Ae.hirsitus* | 3b |
| 30-06-23 | Lamu | Mokowe | S2 | 3 | LAM/413 | Light Trap | F | 3 | *Cx.pipiens* | 3b |
| 30-06-23 | Lamu | Mokowe | S2 | 3 | LAM/414 | Light Trap | F | 1 | *Cx.annulioris* | 3b |
| 30-06-23 | Lamu | Mokowe | S2 | 3 | LAM/415 | Light Trap | F | 1 | *Ae.metallicus* | 3b |
| 30-06-23 | Lamu | Mokowe | S2 | 3 | LAM/416 | Light Trap | F | 5 | *Ae.pembaensis* | 3b |
| 30-06-23 | Lamu | Mokowe | S2 | 3 | LAM/417 | Light Trap | F | 6 | *Ae.vittatus* | 3b |
| 30-06-23 | Lamu | Mokowe | S2 | 3 | LAM/418 | Light Trap | M | 3 | *Ae.vittatus* | 3b |
| 30-06-23 | Lamu | Mokowe | S2 | 3 | LAM/419 | Light Trap | M | 6 | *Ae.aegypti* | 3b |
| 30-06-23 | Lamu | Mokowe | S2 | 3 | LAM/420 | Light Trap | F | 25 | *Ae.aegypti* | 3b |
| 30-06-23 | Lamu | Mokowe | S2 | 3 | LAM/421 | Light Trap | F | 2 | *Ae.aegypti* | 3b |
| 29-06-23 | Lamu | Shela | S2 | 2 | LAM/423 | Light Trap | F | 2 | *Ae.hirsitus* | 3b |
| 29-06-23 | Lamu | Shela | S2 | 2 | LAM/423 | Light Trap | F | 2 | *Cx.pipiens* | 3b |
| 29-06-23 | Lamu | Shela | S2 | 2 | LAM/423 | Light Trap | F | 4 | *Ae.pembaensis* | 3b |
| 30-06-23 | Lamu | Mokowe | S2 | 2 | LAM/425 | Light Trap | F | 1 | *Ae.hirsitus* | 3b |
| 30-06-23 | Lamu | Mokowe | S2 | 2 | LAM/426 | Light Trap | F | 11 | *Ae.pembaensis* | 3b |
| 30-06-23 | Lamu | Mokowe | S2 | 2 | LAM/427 | Light Trap | M | 12 | *Ae.vittatus* | 3b |
| 30-06-23 | Lamu | Mokowe | S2 | 2 | LAM/428 | Light Trap | F | 17 | *Ae.vittatus* | 3b |
| 30-06-23 | Lamu | Mokowe | S2 | 2 | LAM/429 | Light Trap | M | 8 | *Ae.aegypti* | 3b |
| 30-06-23 | Lamu | Mokowe | S2 | 2 | LAM/430 | Light Trap | F | 25 | *Ae.aegypti* | 3b |
| 29-06-23 | Lamu | Shela | S4 | 4 | LAM/431 | Light Trap | F | 2 | *Ae.quasiunivittatus* | 3b |
| 29-06-23 | Lamu | Shela | S4 | 4 | LAM/432 | Light Trap | F | 4 | *Cx.pipiens* | 3b |
| 29-06-23 | Lamu | Shela | S4 | 1 | LAM/433 | Light Trap | F | 2 | *Ae.quasiunivittatus* | 3b |
| 29-06-23 | Lamu | Shela | S4 | 1 | LAM/434 | Light Trap | F | 6 | *Cx.pipiens* | 3b |
| 29-06-23 | Lamu | Shela | S4 | 1 | LAM/435 | Light Trap | F | 8 | *Ae.pembaensis* | 3b |
| 29-06-23 | Lamu | Shela | S4 | 3 | LAM/436 | Light Trap | F | 4 | *Ae.hirsitus* | 3b |
| 29-06-23 | Lamu | Shela | S4 | 3 | LAM/437 | Light Trap | F | 9 | *Cx.pipiens* | 3b |
| 29-06-23 | Lamu | Shela | S4 | 3 | LAM/438 | Light Trap | F | 8 | *Ae.pembaensis* | 3b |
| 29-06-23 | Lamu | Shela | S4 | 6 | LAM/439 | Light Trap | F | 1 | *Ae.aegypti* | 3b |
| 29-06-23 | Lamu | Shela | S4 | 6 | LAM/440 | Light Trap | F | 11 | *Cx.pipiens* | 3b |
| 29-06-23 | Lamu | Shela | S4 | 6 | LAM/441 | Light Trap | F | 3 | *Ae.pembaensis* | 3b |
| 29-06-23 | Lamu | Shela | S4 | 5 | LAM/442 | Light Trap | F | 3 | *Cx.pipiens* | 3b |
| 29-06-23 | Lamu | Shela | S4 | 5 | LAM/443 | Light Trap | F | 5 | *Ae.pembaensis* | 4b |
| 29-06-23 | Lamu | Shela | S4 | 5 | LAM/444 | Light Trap | F | 2 | *Ae.hirsitus* | 4b |
| 29-06-23 | Lamu | Mokowe | S2 | 3 | LAM/445 | Light Trap | F | 2 | *Ae.metallicus* | 4b |
| 29-06-23 | Lamu | Mokowe | S2 | 3 | LAM/446 | Light Trap | F | 3 | *Cx.pipiens* | 4b |
| 29-06-23 | Lamu | Mokowe | S2 | 3 | LAM/447 | Light Trap | F | 2 | *Coq.aurites* | 4b |
| 29-06-23 | Lamu | Mokowe | S2 | 3 | LAM/448 | Light Trap | F | 4 | *Coq.metallicus* | 4b |
| 29-06-23 | Lamu | Mokowe | S2 | 3 | LAM/449 | Light Trap | F | 2 | *Ae.pembaensis* | 4b |
| 29-06-23 | Lamu | Mokowe | S2 | 3 | LAM/450 | Light Trap | M | 7 | *Ae.aegypti* | 4b |
| 29-06-23 | Lamu | Mokowe | S2 | 3 | LAM/451 | Light Trap | F | 25 | *Ae.aegypti* | 4b |
| 29-06-23 | Lamu | Mokowe | S2 | 3 | LAM/452 | Light Trap | F | 3 | *Ae.aegypti* | 4b |
| 29-06-23 | Lamu | Mokowe | S2 | 3 | LAM/453 | Light Trap | F | 21 | *Mn.africanus* | 4b |
| 29-06-23 | Lamu | Mokowe | S2 | 3 | LAM/454 | Light Trap | F | 25 | *Mn.uniformis* | 4b |
| 29-06-23 | Lamu | Mokowe | S2 | 3 | LAM/455 | Light Trap | F | 25 | *Mn.uniformis* | 4b |
| 29-06-23 | Lamu | Mokowe | S2 | 3 | LAM/456 | Light Trap | F | 18 | *Mn.uniformis* | 4b |
| 29-06-23 | Lamu | Mokowe | S2 | 2 | LAM/457 | Light Trap | F | 2 | *Ae.tricholabis* | 4b |
| 29-06-23 | Lamu | Mokowe | S2 | 2 | LAM/458 | Light Trap | F | 1 | *Cx.annulioris* | 4b |
| 29-06-23 | Lamu | Mokowe | S2 | 2 | LAM/459 | Light Trap | F | 1 | *Ae.simpsoni* | 4b |
| 29-06-23 | Lamu | Mokowe | S2 | 2 | LAM/460 | Light Trap | F | 1 | *Coq.aurites* | 4b |
| 29-06-23 | Lamu | Mokowe | S2 | 2 | LAM/461 | Light Trap | F | 1 | *Ae.pembaensis* | 4b |
| 29-06-23 | Lamu | Mokowe | S2 | 2 | LAM/462 | Light Trap | F | 4 | *Cx.pipiens* | 4b |
| 29-06-23 | Lamu | Mokowe | S2 | 2 | LAM/463 | Light Trap | M | 3 | *Ae.aegypti* | 4b |
| 29-06-23 | Lamu | Mokowe | S2 | 2 | LAM/464 | Light Trap | F | 18 | *Ae.aegypti* | 4b |
| 29-06-23 | Lamu | Mokowe | S2 | 2 | LAM/465 | Light Trap | F | 12 | *Mn.africanus* | 4b |
| 29-06-23 | Lamu | Mokowe | S2 | 2 | LAM/466 | Light Trap | F | 25 | *Mn.uniformis* | 4b |
| 29-06-23 | Lamu | Mokowe | S2 | 2 | LAM/467 | Light Trap | F | 25 | *Mn.uniformis* | 4b |
| 29-06-23 | Lamu | Mokowe | S2 | 2 | LAM/468 | Light Trap | F | 10 | *Mn.uniformis* | 4b |
| 29-06-23 | Lamu | Mokowe | S2 | 4 | LAM/469 | Light Trap | F | 1 | *Ae.tricholabis* | 4b |
| 29-06-23 | Lamu | Mokowe | S2 | 4 | LAM/470 | Light Trap | F | 1 | *Ae.pembaensis* | 4b |
| 29-06-23 | Lamu | Mokowe | S2 | 4 | LAM/471 | Light Trap | F | 1 | *Coq.metallicus* | 4b |
| 29-06-23 | Lamu | Mokowe | S2 | 4 | LAM/472 | Light Trap | F | 13 | *Cx.pipiens* | 4b |
| 29-06-23 | Lamu | Mokowe | S2 | 4 | LAM/473 | Light Trap | M | 14 | *Ae.aegypti* | 4b |
| 29-06-23 | Lamu | Mokowe | S2 | 4 | LAM/474 | Light Trap | F | 25 | *Ae.aegypti* | 4b |
| 29-06-23 | Lamu | Mokowe | S2 | 4 | LAM/475 | Light Trap | F | 25 | *Ae.aegypti* | 4b |
| 29-06-23 | Lamu | Mokowe | S2 | 4 | LAM/476 | Light Trap | F | 6 | *Ae.aegypti* | 4b |
| 29-06-23 | Lamu | Mokowe | S2 | 4 | LAM/477 | Light Trap | F | 23 | *Mn.africanus* | 4b |
| 29-06-23 | Lamu | Mokowe | S2 | 4 | LAM/478 | Light Trap | F | 25 | *Mn.uniformis* | 4b |
| 29-06-23 | Lamu | Mokowe | S2 | 4 | LAM/479 | Light Trap | F | 13 | *Mn.uniformis* | 4b |
| 29-06-23 | Lamu | Mokowe | S2 | 1 | LAM/480 | Light Trap | F | 1 | *Ae.metallicus* | 4b |
| 29-06-23 | Lamu | Mokowe | S2 | 1 | LAM/481 | Light Trap | F | 2 | *Cx.univittatus* | 4b |
| 29-06-23 | Lamu | Mokowe | S2 | 1 | LAM/482 | Light Trap | F | 2 | *Ae.pembaensis* | 4b |
| 29-06-23 | Lamu | Mokowe | S2 | 1 | LAM/483 | Light Trap | F | 2 | *Cx.pipiens* | 4b |
| 29-06-23 | Lamu | Mokowe | S2 | 1 | LAM/484 | Light Trap | F | 1 | *Cx.annulioris* | 4b |
| 29-06-23 | Lamu | Mokowe | S2 | 1 | LAM/485 | Light Trap | F | 1 | *Ae.tricholabis* | 4b |
| 29-06-23 | Lamu | Mokowe | S2 | 1 | LAM/486 | Light Trap | M | 6 | *Ae.aegypti* | 4b |
| 29-06-23 | Lamu | Mokowe | S2 | 1 | LAM/487 | Light Trap | F | 25 | *Ae.aegypti* | 4b |
| 29-06-23 | Lamu | Mokowe | S2 | 1 | LAM/488 | Light Trap | F | 8 | *Ae.aegypti* | 4b |
| 29-06-23 | Lamu | Mokowe | S2 | 1 | LAM/489 | Light Trap | F | 18 | *Mn.africanus* | 4b |
| 29-06-23 | Lamu | Mokowe | S2 | 1 | LAM/490 | Light Trap | F | 25 | *Mn.uniformis* | 4b |
| 29-06-23 | Lamu | Mokowe | S2 | 1 | LAM/491 | Light Trap | F | 12 | *Mn.uniformis* | 4b |
